# Embryonic progenitor pools generate diversity in fine-scale excitatory cortical subnetworks

**DOI:** 10.1038/s41467-019-13206-1

**Published:** 2019-11-19

**Authors:** Tommas J. Ellender, Sophie V. Avery, Kashif Mahfooz, Jakub Scaber, Alexander von Klemperer, Sophie L. Nixon, Matthew J. Buchan, Joram J. van Rheede, Aleksandra Gatti, Cameron Waites, Hania J. Pavlou, David Sims, Sarah E. Newey, Colin J. Akerman

**Affiliations:** 1 Department of Pharmacology, University of Oxford, Mansfield Road, Oxford, OX1 3QT UK; 2 MRC Computational Genomics Analysis and Training Programme (CGAT), MRC WIMM Centre for Computational Biology, MRC Weatherall Institute of Molecular Medicine, Oxford, OX3 9DS UK

**Keywords:** Developmental biology, Neuroscience, Neural circuits

## Abstract

The mammalian neocortex is characterized by a variety of neuronal cell types and precise arrangements of synaptic connections, but the processes that generate this diversity are poorly understood. Here we examine how a pool of embryonic progenitor cells consisting of apical intermediate progenitors (aIPs) contribute to diversity within the upper layers of mouse cortex. In utero labeling combined with single-cell RNA-sequencing reveals that aIPs can generate transcriptionally defined glutamatergic cell types, when compared to neighboring neurons born from other embryonic progenitor pools. Whilst sharing layer-associated morphological and functional properties, simultaneous patch clamp recordings and optogenetic studies reveal that aIP-derived neurons exhibit systematic biases in both their intralaminar monosynaptic connectivity and the post-synaptic partners that they target within deeper layers of cortex. Multiple cortical progenitor pools therefore represent an important factor in establishing diversity amongst local and long-range fine-scale glutamatergic connectivity, which generates subnetworks for routing excitatory synaptic information.

## Abstract (web summary)

Glutamatergic neurons in the mammalian cortex are born from a heterogeneous pool of embryonic progenitors, however, it is unclear how these different progenitors contribute to diversity within the mature cortex. In this study, authors combine in utero progenitor labeling techniques with targeted Patch-Seq methods and high resolution synaptic circuit mapping in the mature mouse cortex to show that intermediate progenitors can generate restricted sets of transcriptomically-defined glutamatergic neurons that have distinct patterns of local and long-range synaptic connections.

## Introduction

The mammalian neocortex is a laminar structure containing numerous cell types that are precisely interconnected and assembled into neural circuits^1,2^. Understanding how this neuronal diversity arises and what principles govern the formation of the precise synaptic connections are major challenges for neuroscience. Cortical excitatory glutamatergic neurons include both pyramidal neurons and spiny stellate neurons, which can be further divided into distinct cell types according to laminar, morphological, electrophysiological, and transcriptomic criteria^3–8^. A further distinguishing feature is the fine-scale synaptic connectivity of excitatory cortical neurons, which has been associated with multiple factors including whether neurons share the same laminar position, the same cortical and subcortical post-synaptic targets^9–14^, similar sensory response properties^15^ or a common lineage^16–19^.

All excitatory cortical neurons are born during embryonic development from a heterogeneous population of neural progenitors located in the ventricular and subventricular proliferative zones (VZ and SVZ) of the pallium^20–26^. In the mouse for example, the principal class of cortical progenitors are radial glial cells, which reside within the VZ and undergo asymmetric, self-renewing cell-divisions to generate either neurons or intermediate progenitor cells^20,24,27–29^. Intermediate progenitors are associated with symmetrical neurogenic divisions that produce two post-mitotic daughter neurons and are therefore considered transient amplifying progenitors that augment cortical neurogenesis. In addition to radial glial cells, the mouse VZ contains so-called apical intermediate progenitors (aIPs; also referred to as short neural precursors^21,22,27,29^), which have been shown to contribute to the upper cortical layers, particularly L4 and L2/3, and to differ from other VZ progenitors in terms of their morphology and cell cycle behavior^21,22^. Meanwhile, the SVZ progenitor pool includes basal intermediate progenitor cells and outer radial glia, whose relative numbers vary across species, but are also thought to derive from radial glia in the VZ^20,23,24,28–34^.

An important question is whether intermediate progenitor populations contribute to diversity within the mature cortex. One possibility is that intermediate progenitors solely provide an expansion of cortical layers, by amplifying the neuronal output of the overall excitatory progenitor pool^27^. Alternatively, intermediate progenitors may also contribute to the cellular and circuit diversity within cortex, by conferring specific identities to their neuronal progeny^4,35^. Here we examine how aIPs contribute to diversity within the upper layers of mouse somatosensory cortex. Using in utero labeling methods to preferentially target aIPs, single-cell RNA-sequencing, simultaneous patch-clamp recordings and optogenetic studies, we characterize excitatory cortical neurons derived from aIPs and compare them with neighboring excitatory neurons within the same layer, but derived from other progenitor pools. Our data reveal that aIPs give rise to a restricted population of upper layer cortical neurons, which can share many functional and morphological properties with adjacent excitatory neurons derived from different progenitor pools, but differ significantly in terms of their post-synaptic partners. Compared with neighboring neurons within the same layer, aIP-derived neurons show distinct arrangements of local intralaminar and extralaminar connectivity, which implicates aIPs in the generation of fine-scale excitatory subnetworks for differentially routing cortical information.

## Results

### Progenitor pools generate specific cortical neuron types

**Fig. 1:**
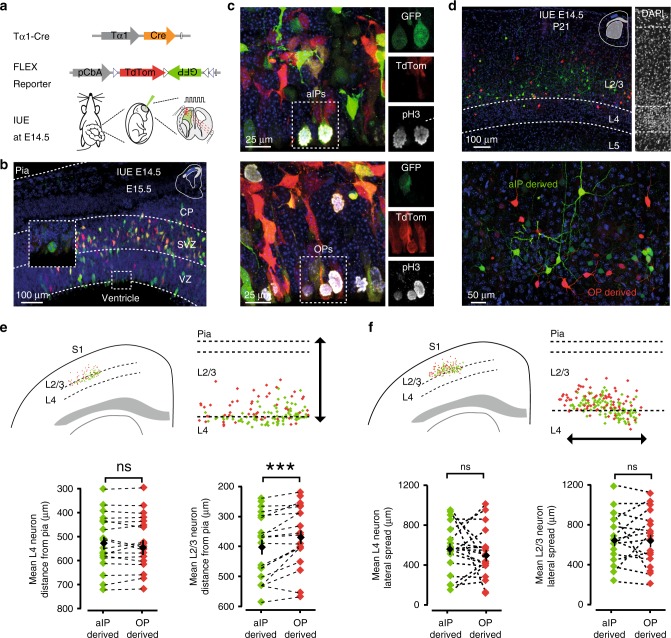
In utero labeling of neurons derived from different progenitor pools. **a** In utero electroporation (IUE) was used to deliver a Tα1-Cre and two-color CβA-FLEx reporter plasmid into mouse cortical progenitor cells. **b** 24 h later, GFP-expressing cells in the VZ exhibited properties of apical intermediate progenitor cells (aIPs), including a lack of basal process during mitosis (inset). Cells expressing only TdTomato exhibited properties associated with other progenitors (OPs). **c** Actively dividing aIPs (top) and OPs (bottom) were positive for the mitotic marker phospho-histone H3. **d** One month after IUE (P21), L4 and L2/3 neurons within somatosensory cortex (top) could be distinguished as either aIP-derived (GFP^+^) or OP-derived (TdTomato^+^; bottom). Cortical layers were delineated with DAPI staining. **e** Representative postnatal brain slice and corresponding scatter plot (top) indicating the positions of aIP-derived (green) and OP-derived (red) neurons. Within L4, the mean radial position of the two neuronal populations was similar (bottom; *p* = 0.97, paired *t*-test, *n* = 19). Within L2/3, the mean radial position was deeper for aIP-derived neurons compared with the OP-derived population (*p* = 0.0005, paired *t*-test, *n* = 19). **f** Example brain slice and scatter plot indicating the lateral spread of labeled neurons (top). The lateral extent of aIP- and OP-derived neurons was not different within L4 or L2/3 (bottom; *p* = 0.45 and *p* = 0.97, respectively, paired *t*-test). Error bars represent standard error of the mean. Source data are provided as a Source Data file

To investigate the relationship between progenitor pool and excitatory cortical neuronal identity, we pulse-labeled two populations of dividing progenitor cells at gestation age E14.5 in C57BL/6 mouse embryos, when glutamatergic neurons in the upper cortical layers are being generated. We made use of the fact that the tubulin alpha1 (*Tα1*) promoter can be used to preferentially label apical intermediate progenitors (aIPs) within the VZ^21,22^. This intermediate progenitor population has been distinguished from other progenitors (OPs), which are thought to include radial glial cells within the VZ, and outer radial glia and basal intermediate progenitor cells within the SVZ^20,24,28,29,32–34^. We labeled the progenitor populations using in utero electroporation (IUE), which has been shown to target actively dividing cells in the VZ, located close to the ventricular surface^22^. Two DNA constructs were electroporated: a Tα1-Cre construct in which Cre recombinase is under the control of a portion of the *Tα1* promoter^22^, and a CβA-FLEx reporter construct that incorporates a flexible excision (FLEx) cassette where Cre recombination permanently switches expression from TdTomato fluorescent protein to enhanced green fluorescent protein (GFP)^23^ (Fig. 1a–d; Supplementary Fig. 1; see Methods). Consistent with earlier work, 24 h after IUE the Tα1-Cre construct preferentially labeled a GFP^+^ progenitor population that exhibited characteristics of aIPs^21,22^. We replicated previous observations that, compared with OPs, the GFP^+^ aIPs lacked a basal process during metaphase, exhibited short ascending processes during their cell cycle, and represented a larger proportion of the VZ progenitors at E14.5 compared with E13.5 (Supplementary Figs. 1 and 3)^21^. At 24 h post-IUE, the majority of TdTomato^+^ progenitors exhibited a basal process that reached the cortical surface, consistent with a radial glial cell morphology. Control experiments confirmed that the recombination process occurred during embryonic development, accurately reflected the promoter driving Cre, and resulted in the stable labeling of cortical neurons into adulthood (Supplementary Figs. 2, 3). Therefore, at the point of labeling, this strategy marked a progenitor population enriched for aIPs, and a population of concurrently dividing OPs. Postnatally, labeled neurons could then be assigned as having derived from one of these two progenitor pools. We refer to the GFP^+^ and TdTomato^+^ progeny of these cells as “aIP-derived” and “OP-derived”, respectively.

IUE was targeted to the VZ region that gives rise to neurons in primary somatosensory cortex (S1), such that when animals were allowed to survive postnatally, many fluorescently labeled neurons were observed in L4 and L2/3 of S1 (Fig. 1d). GFP^+^ aIP-derived neurons were interspersed with TdTomato^+^ OP-derived neurons, and the two populations had overlapping vertical and horizontal distributions (Fig. 1e, f). Across animals, the proportion of aIP-derived neurons in L4 and L2/3 was 37.7 ± 3.9% and 62.3 ± 3.9%, respectively (mean ± SEM; *n* = 19). While the proportion of OP-derived neurons in L4 and L2/3 was 34.8 ± 5.5% and 65.2 ± 5.5%, respectively. Of the labeled cells in L4, 64.7 ± 6.4% were aIP-derived, 35.3 ± 6.3% were OP-derived. While in L2/3, 62.5 ± 5.2% of the labeled cells were aIP-derived and 37.5 ± 5.2% were OP-derived. The mean radial location of the two populations was comparable in L4, while there was a tendency for the soma of aIP-derived neurons to reside somewhat deeper within L2/3 (Fig. 1e). This replicates an earlier observation regarding the progeny of aIPs compared with OPs, which has been linked to the degree of lineage heterogeneity^22^. No difference was observed in the lateral distribution of labeled soma in L4 or L2/3 (Fig. 1f).

**Fig. 2:**
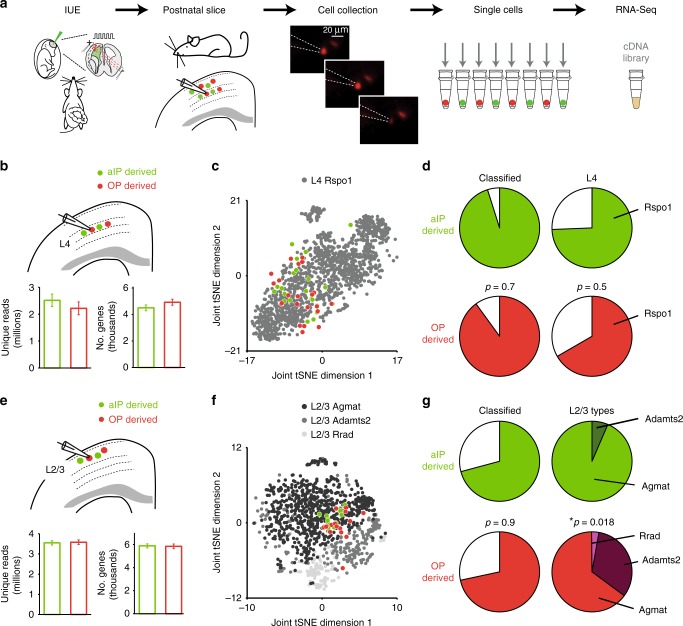
aIPs contribute to specific upper cortical layer neuron types. **a** Neighboring aIP-derived (GFP^+^) and OP-derived (TdTomato^+^) neurons were isolated alternately from S1 in acutely prepared postnatal brain slices. Batches of neurons from the same cortical layer were subjected to single-cell RNA-seq. **b** Within L4, aIP- and OP-derived neurons (top; *n* = 85 and 77 across P10 and P30, respectively) generated comparable numbers of unique reads (bottom; *p* = 0.37, *t*-test) and detected genes (*p* = 0.19, *t*-test). **c** Example tSNE plot shows that aIP- and OP-derived L4 neurons at P30 distribute similarly amongst “L4 *Rspo1* neurons”—the single L4 excitatory neuron type identified in a recently published transcriptomic cell type atlas of adult mouse primary visual cortex (gray; ref. ^3^). **d** Similar proportions of L4 neurons classified at P30 (left; *p* = 0.7, Chi Square test, *n* = 41 aIP- and 40 OP-derived) and, of these, a similar proportion were identified as L4 *Rspo* cells (right; *p* = 0.5, Chi Square test). **e** Within L2/3, aIP- and OP-derived neurons (top; *n* = 110 and 106 neurons across P10 and P30, respectively) generated comparable numbers of unique reads (bottom; *p* = 0.86, *t*-test) and detected genes (*p* = 0.88, *t*-test). **f** Example tSNE plot showing aIP- and OP-derived L2/3 neurons at P30, combined with the three L2/3 excitatory neuron types from adult mouse primary visual cortex: “L2/3 *Agmat*”, “L2/3 *Adamts2*”, and “L2/3 *Rrad*” neurons (gray data; ref. ^3^). **g** While a similar proportion of P30 neurons classified (left; *p* = 0.9, Chi Square test, *n* = 51 aIP- and 48 OP-derived), aIP-derived neurons were significantly more restricted in terms of cell types (right). aIP-derived neurons were almost exclusively of the *Agmat* cell class, while OP-derived included *Agmat*, *Adamts2*, and *Rrad* L2/3 neurons (*p* = 0.018, Chi Square test). Error bars represent standard error of the mean. Source data are provided as a Source Data file

Recent single-cell RNA-seq analysis of mouse neocortex has provided a transcriptomic-based taxonomy of cortical cell types, comprising over 100 distinct cell classes^3^. This includes a single L4 excitatory neuron type, referred to as “*Rspo1*” glutamatergic neurons, and three subtypes of L2/3 excitatory neuron, referred to as “*Agmat*”, “*Adamts2*”, and “*Rrad*” glutamatergic neurons^3^. To investigate whether the aIP and OP progenitor pools generate these transcriptionally defined subtypes, we used an adapted version of the Patch-seq technique^36,37^ in which neighboring TdTomato^+^ and GFP^+^ neurons were collected alternately from a single cortical layer within acutely prepared postnatal brain slices (Fig. 2a; see Methods). From L4, we analyzed single-cell libraries from a total of 90 aIP-derived neurons and 90 OP-derived neurons across two postnatal ages (P10 and P30). Following removal of low-quality cells (~10%; see Methods and Supplementary Fig. 4), analysis was performed on 85 aIP-derived and 77 OP-derived L4 neurons. Each cell had ~2.40 million uniquely mapping reads and showed detection of 5000 genes, which was comparable for the aIP- and OP-derived populations (Fig. 2b). After combining our data with the published dataset of L4 glutamatergic neurons (*n* = 1401 L4 *Rspo1* neurons^3^), initial analyses using a dimensionality reduction method (t-distributed Stochastic Neighbor Embedding; tSNE), suggested that the aIP- and OP-derived populations showed similar heterogeneity (Fig. 2c).

To assess how our L4 neurons compared with the previously described neuronal cells, we used a classification method based on the unique expression profiles of the 115 cortical cell types reported for mouse visual cortex^3^. This enabled us to assign each of our cells to its most likely cell class, contingent on passing a confidence threshold (see Methods). Our first observation was that cell classification was more successful at P30 than at P10 (Supplementary Fig. 5; 93% cells classified vs. 14% cells classified, respectively; *p* < 0.0001 Chi Square test), suggesting that cell identity may emerge with age. Furthermore, we found that similar proportions of aIP- and OP-derived L4 neurons were successfully classified at P30, and of these, a comparable proportion were identified as *Rspo1* L4 neurons (Fig. 2d; 74 and 65%, *p* = 0.5, Chi Square test, *n* = 39 and 36). These data indicate that aIPs and OPs contribute similarly to *Rspo1* L4 neurons within mouse S1.

In separate experiments we collected individual L2/3 neurons from S1. Following removal of low-quality cells, analysis and cell classification was performed on 109 aIP-derived and 105 OP-derived L2/3 neurons (across P10 and P30). Each cell had ~3.6 million uniquely mapping reads and resulted in the detection of 5900 genes, which was comparable for the two populations (Fig. 2e). Exploratory analyses after combining our L2/3 data and the published dataset of L2/3 glutamatergic cortical neurons (*n* = 981 L2/3 neurons^3^), suggested that the OP-derived population was more heterogeneous than the aIP-derived population (Fig. 2f). This was confirmed when we classified our L2/3 neurons against known cortical cell types. We found that a comparable proportion of aIP- and OP-derived L2/3 neurons were successfully assigned to a cortical cell type (Fig. 2g). However, the breakdown of the L2/3 subtypes was very different. OP-derived neurons comprised a mixture of *Agmat* (65%), *Adamts2* (32%), and *Rrad* (3%). In contrast, the aIP-derived neurons were significantly more homogenous, as they were comprised of almost entirely the *Agmat* class (94%) and only a small proportion of *Adamts2* (6%). The distribution of cell types derived from aIPs was statistically distinct to those derived from OPs (*p* = 0.018, Chi Square test, *n* = 34 and 31 classified neurons, respectively). This was supported by comparisons to the proportion of the different neuron types previously reported amongst the general L2/3 population^3^: 68% *Agmat*, 21% *Adamts2*, and 11% *Rrad* (*n* = 981). Statistical analyses revealed that while the OP-derived neurons did not differ to the general population (*p* = 0.15, Chi Square test), the proportions of the aIP-derived L2/3 neurons were significantly different to those amongst the general population (*p* = 0.009, Chi Square test). Therefore, aIPs show a more restricted output by primarily contributing to a particular transcriptionally defined L2/3 neuronal sub-type.

### Local intralaminar connectivity reflects progenitor pool

**Fig. 3:**
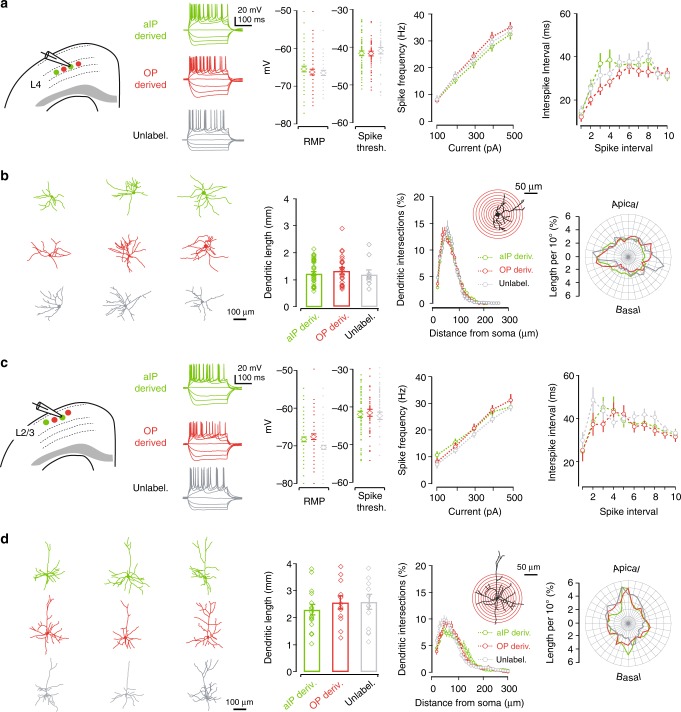
Neuronal excitability and morphology do not differ by progenitor pool. **a** aIP- and OP-derived L4 neurons exhibited comparable intrinsic membrane properties, including resting membrane potential (RMP; *p* = 0.26), spike threshold (*p* = 0.52) and spiking patterns to injected current (spike frequency: *p* = 0.27; 1st interspike interval: *p* = 0.22, Mann–Whitney U test in each case). **b** Anatomical reconstruction of aIP- and OP-derived L4 spiny stellate neurons revealed comparable dendritic lengths, dendritic complexity as assessed by Scholl analysis, and dendritic polarity (*p* = 0.28, *p* = 0.74, and *p* = 0.51, ANOVA). **c** Within L2/3, aIP- and OP-derived neurons exhibited comparable membrane properties, including RMP (*p* = 0.89), spike threshold (*p* = 0.49), and spiking patterns to injected current (spike frequency: *p* = 0.66; 1st interspike interval: *p* = 0.65, Mann–Whitney U test in each case). **d** aIP- and OP-derived L2/3 neurons exhibited pyramidal morphologies with comparable dendritic lengths, complexity, and polarity (*p* = 0.39, *p* = 0.82, and *p* = 0.99, ANOVA). Error bars represent standard error of the mean. Source data are provided as a Source Data file

We next used the same IUE labeling strategy to characterize the intrinsic membrane properties and basic cell morphology of aIP- and OP-derived neurons within each cortical layer. In acute brain slices prepared at ~4 weeks of age, fluorescently labeled neurons were targeted for whole-cell patch-clamp recordings, filled with biocytin and their morphologies reconstructed (Fig. 3 and Supplementary Table 1). L4 neurons derived from aIPs and OPs were found to exhibit comparable behavior in terms of their intrinsic excitability, including resting membrane potential, spike threshold and spiking patterns to injected current (Fig. 3a). Meanwhile, quantitative morphological analysis revealed that aIP- and OP-derived neurons displayed spiny stellate morphologies with comparable dendritic lengths, complexity and polarity (Fig. 3b). Similarly, L2/3 neurons derived from aIPs and OPs exhibited comparable intrinsic excitability and morphologies to one another. The two L2/3 populations exhibited comparable membrane potential and spiking properties (Fig. 3c and Supplementary Table 1), and the anatomical reconstructions revealed that aIP- and OP-derived neurons had pyramidal morphologies with comparable dendritic lengths, complexity and polarity (Fig. 3d). These data indicate that aIP-derived neurons share many basic, layer-related cellular properties, with neighboring neurons derived from OPs.

**Fig. 4:**
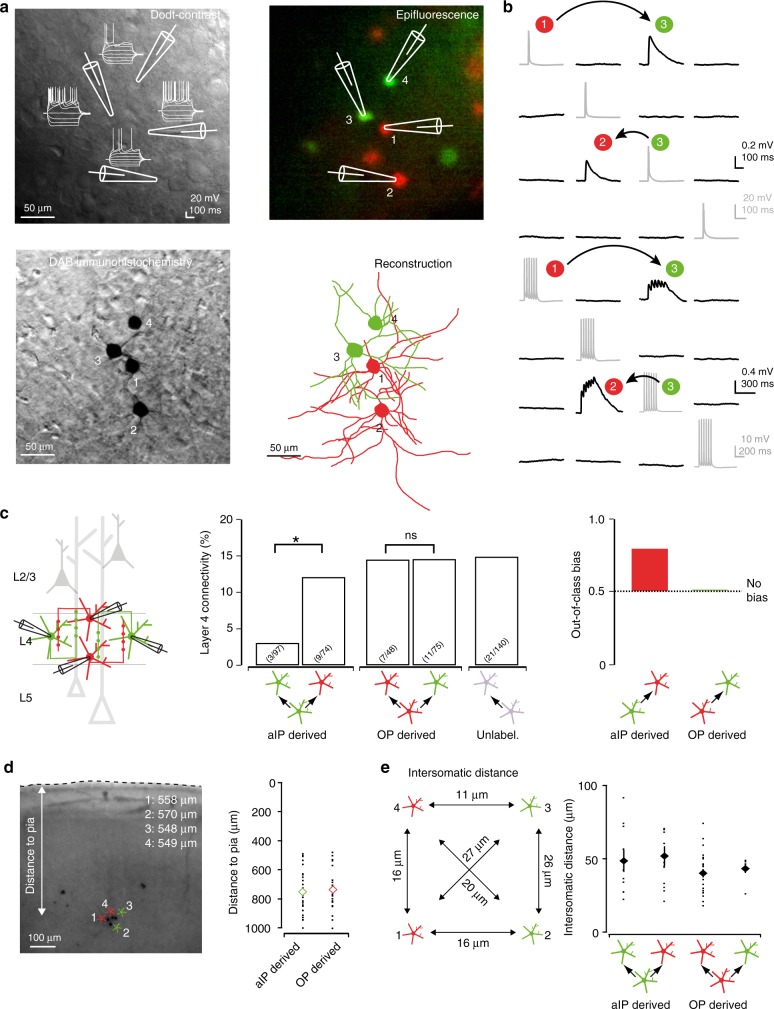
aIP-derived L4 neurons exhibit selective intralaminar connections. **a** Quadruplet whole-cell patch-clamp recording from aIP- and OP-derived neurons in L4 of S1. **b** Intralaminar connectivity was assessed with single (top) and trains (bottom) of pre-synaptic action potentials. **c** aIP-derived L4 spiny stellate neurons exhibited a higher probability of connecting to neighboring L4 neurons that were derived from OPs (aIP to aIP: 3.1%, aIP to OP: 12.2%, OP to OP: 14.6%, OP to aIP: 14.7%; aIP to OP: *p* = 0.03, Fisher’s exact test). This represents an ‘out-of-class’ connectivity bias for aIP-derived neurons, where a value of 0.5 indicates no bias. **d** Recorded aIP- and OP-derived neurons had comparable distances from the pial surface (*p* = 0.7, *t*-test, *n* = 28 and 26). **e** Intersomatic distances were similar for each category of cell-cell comparison (*p* = 0.5, Kruskal–Wallis test, *n* = 10, 12, 23, and 7). Error bars represent standard error of the mean. Source data are provided as a Source Data file

Previous work has shown that the choice of post-synaptic partners is a distinguishing feature of excitatory cortical neurons. For example, differences in local monosynaptic connectivity have been linked to whether neurons share the same laminar position, response properties, or are part of the same excitatory subnetwork for routing excitatory synaptic information^9–15,38^. To examine whether aIP-derived cortical neurons differ in their arrangements of post-synaptic partners, we performed simultaneous targeted whole-cell patch-clamp recordings (quadruplet/triplet/paired recordings) and assessed synaptic connectivity amongst aIP-derived, OP-derived and unlabeled S1 neurons. Within L4, we studied 434 potential intralaminar connections by eliciting action potentials in each putative pre-synaptic neuron and determining whether a response occurred in the putative post-synaptic neuron (Fig. 4a, b). Of the potential connections that were sampled, 51 were found to be monosynaptically connected (delay 1.84 ± 0.14 ms), reflecting an overall average connection probability of 11.8%. However, when analyzing the data according to progenitor pool of origin, we found that aIP-derived neurons were significantly more likely to synapse onto OP-derived neurons than to other aIP-derived neurons, at a ratio of 12.2% to 3.1% (*p* = 0.03, Fisher’s exact test; Fig. 4c).

In contrast, OP-derived neurons were no more likely to connect to aIP-derived neurons than to other OP-derived neurons (OP to aIP: 14.7% and OP to OP: 14.6%, *p* = 1.00, Fisher’s exact test). This relationship between progenitor source and intralaminar connectivity could be captured as an ‘out-of-class’ bias, where a value of 0.5 means the pre-synaptic neurons exhibit no preference based on the post-synaptic cell’s progenitor pool, and a value of 1.0 means the pre-synaptic neurons connect exclusively with post-synaptic cells derived from a different progenitor pool. While the out-of-class connectivity bias was 0.5 for OP-derived L4 neurons, the corresponding value for aIP-derived neurons was 0.8 (Fig. 4c). These differences in connectivity were not associated with differences in the properties of the synaptic connections (Supplementary Table 2), the somatic location within L4 (Fig. 4d), or the distances between the recorded neurons (Fig. 4e).

**Fig. 5:**
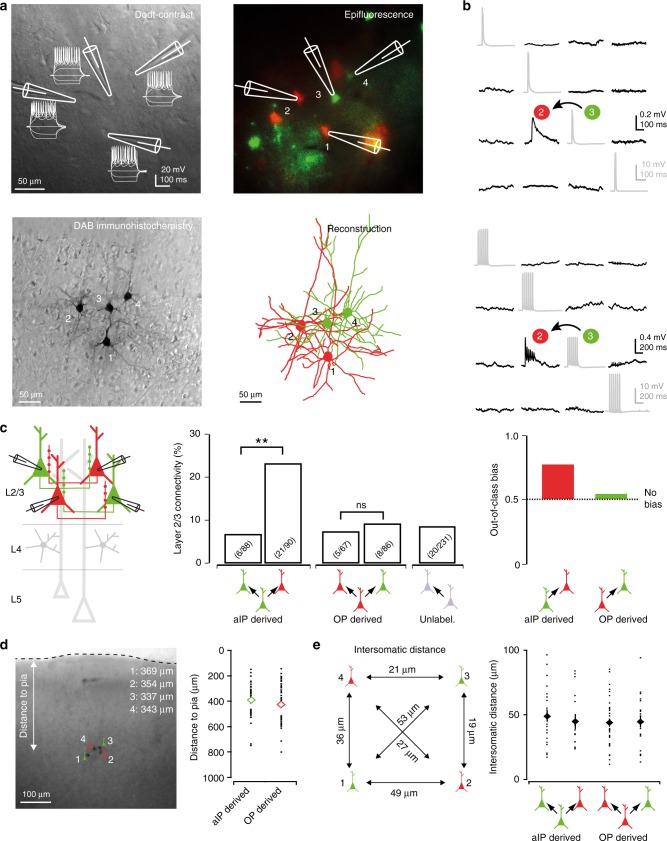
aIP-derived L2/3 neurons exhibit specific intralaminar connections. **a** Quadruplet whole-cell patch-clamp recording from aIP-derived and OP-derived neurons in L2/3. **b** Intralaminar connectivity was assessed with single (top) and trains (bottom) of pre-synaptic action potentials. **c** aIP-derived L2/3 pyramidal neurons exhibited a higher probability of connecting to neighboring L2/3 pyramidal neurons derived from OPs, rather than from aIPs (aIP to aIP: 6.8%, aIP to OP: 23.3%, OP to OP: 7.5%, OP to aIP: 9.3%; aIP to OP: *p* = 0.003, Fisher’s exact test), representing an out-of-class connectivity bias for aIP-derived L2/3 neurons. **d** Intralaminar connectivity was sampled without spatial bias, as the recorded aIP- and OP-derived neurons had comparable distances from the pial surface (right; *p* = 0.24, *t*-test, *n* = 43 and 45). **e** Intersomatic distances for each category of cell-cell comparison (right; *p* = 0.8, Kruskal–Wallis test, *n* = 25, 15, 37, and 20). Error bars represent standard error of the mean. Source data are provided as a Source Data file

To establish whether this pattern of local intralaminar connectivity is a general feature of aIP-derived neurons, we examined connectivity between pyramidal neurons in L2/3 (Fig. 5a, b). Of the 562 potential connections that were sampled between L2/3 pyramidal neurons, 60 were found to be monosynaptically connected (delay 2.24 ± 0.19 ms), reflecting an overall average L2/3 pyramidal neuron connection probability of 10.7%. Interestingly, we again found a similar bias in local connectivity that reflected the progenitor of origin. aIP-derived neurons were significantly more likely to synapse onto OP-derived neurons than onto other aIP-derived neurons, at a ratio of 23.3% to 6.8% (*p* = 0.003, Fisher’s exact test; Fig. 5c). This reflected an out-of-class connectivity bias of 0.77 (Fig. 5c). In contrast, OP-derived neurons were no more likely to connect to aIP-derived neurons than to other OP-derived neurons (OP to aIP: 9.3% and OP to OP: 7.5%, *p* = 0.78, Fisher’s exact test), which reflected an out-of-class connectivity bias of 0.55. As in L4, these differences in L2/3 intralaminar connectivity were not associated with differences in the properties of the synaptic connections (Supplementary Table 2), the somatic location within L2/3 (Fig. 5d), or the distances between the recorded neurons (Fig. 5e).

### Translaminar connectivity reflects progenitor pool

**Fig. 6:**
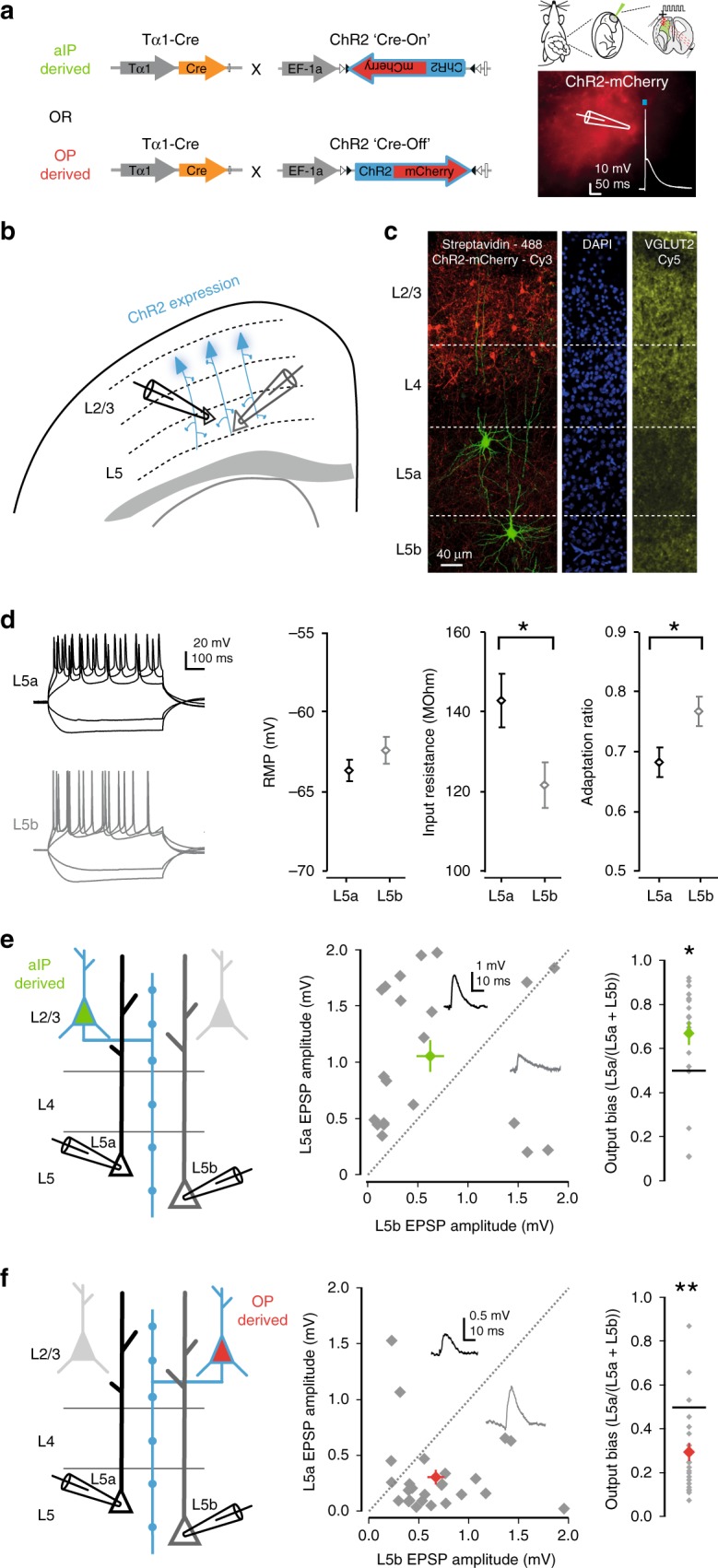
aIPs contribute to diversity in translaminar synaptic circuits. **a** IUE of Tα1-Cre and DIO-ChR2-mCherry (‘Cre-On’) was used to express ChR2 in aIP-derived neurons. Alternatively, Tα1-Cre and DO-ChR2-mCherry (‘Cre-Off’) was used to express ChR2 in OP-derived neurons. **b** Translaminar connectivity was examined at 4 weeks of age by performing simultaneous whole-cell patch-clamp recordings from pairs of L5a and L5b pyramidal neurons, while evoking output from ChR2-expressing L2/3 pyramidal neurons with brief flashes of light. **c** L5 sublayers were delineated post hoc using VGLUT2 immunofluorescence. **d** The identity of L5a (black) and L5b (gray) pyramidal neurons was also confirmed from their intrinsic electrical properties. While their RMP was comparable (*p* = 0.26, *t*-test), L5a pyramidal neurons exhibited higher input resistance (*p* = 0.02, *t*-test) and lower adaptation ratio during spike trains (*p* = 0.02, *t*-test, *n* = 56 and *n* = 51), in line with previous work^40^. **e** aIP-derived L2/3 pyramidal neurons showed a preference to drive L5a, such that larger amplitude responses were consistently recorded in the L5a pyramidal neuron than in the simultaneously recorded L5b pyramidal neuron (*p* = 0.019, Wilcoxon signed rank test, *n* = 21 pairs). **f** In contrast, OP-derived L2/3 pyramidal neurons showed the opposite preference and tended to drive L5b pyramidal neurons more than L5a pyramidal neurons (*p* = 0.001, Wilcoxon signed rank test, *n* = 26 pairs). Error bars represent standard error of the mean. Source data are provided as a Source Data file

aIP-derived neurons in the upper layers of S1 therefore show biases in their selection of post-synaptic target neurons within the same layer, in a manner that reflects the embryonic progenitor type from which the neuron is derived. Such biases in local connectivity are a feature of cortical subnetworks, which are characterized by selective excitatory synaptic targeting both within, and outside of, a cortical layer^9,13,14,39^. To test the hypothesis that aIP-derived neurons also differ in their selection of extralaminar post-synaptic target neurons, we focused on the connection from L2/3 to L5, as L5 represents the main cortical output layer and its sublayers route excitatory synaptic information to different downstream circuits^40^. L5a pyramidal neurons lie more dorsally within L5 and are associated with the direct routing of excitatory synaptic information to other regions of cortex. Meanwhile L5b pyramidal neurons lie more ventrally and have projections that innervate numerous subcortical targets including thalamus, basal ganglia, brainstem sites, and spinal cord^41–43^. Using an optogenetic strategy, we expressed ChR2 in either aIP-derived neurons or OP-derived neurons by IUE of either a Cre-ON or Cre-OFF ChR2 plasmid (see Methods^44^, in combination with the Tα1-Cre plasmid (Fig. 6a). This enabled us to selectively activate axons from either the aIP- or the OP-derived population. We investigated translaminar excitatory synaptic output within the same cortical column by performing simultaneous whole-cell patch-clamp recordings from pairs of L5 pyramidal neurons, one of which was located in L5a and the other in L5b (Fig. 6b–d). Since these experiments involved activating ChR2 fibers originating from a population of L2/3 neurons, a relative difference in post-synaptic response would indicate a net bias in pre-synaptic targeting.

When axons from aIP-derived L2/3 neurons were activated, we observed a pronounced tendency to drive the L5a pyramidal neuron more than the simultaneously recorded L5b neuron. This was captured as an output bias toward L5a of 0.67 ± 0.05, where a value of 0.5 represents equal output (*p* = 0.019, Wilcoxon signed rank test, *n* = 21 pairs; Fig. 6e). This difference was evident regardless of whether the light flash was delivered to the upper half of L2/3, or the lower half of L2/3 (upper output bias: 0.68 ± 0.06, *p* = 0.013, lower output bias: 0.71 ± 0.05, *p* = 0.003, Wilcoxon signed rank test, *n* = 19 and 20 pairs, respectively; Supplementary Fig. 6). In contrast, when axons from OP-derived L2/3 neurons were activated, we observed a pronounced bias in the opposite direction, such that OP-derived L2/3 neurons preferentially drove the L5b pyramidal neuron over the L5a neuron (bias toward L5b of 0.30 ± 0.04; *p* = 0.001, Wilcoxon signed rank test, *n* = 26 pairs**;** Fig. 6f). Again, this difference was evident when the light flash was delivered to either the upper or lower half of L2/3 (upper output bias: 0.29 ± 0.04, *p* = 0.0004, lower output bias: 0.34 ± 0.0064, *p* = 0.002, Wilcoxon signed rank test, *n* = 25 and 22 pairs, respectively; Supplementary Fig. 6). Measurement of the synaptic delays showed that the light-evoked EPSP latency was comparable between the aIP-derived and OP-derived inputs to L5a and L5b neurons (Supplementary Fig. 6). Further, the same biases in output were evident when the data were restricted to recordings in which >90% of the ChR2-expressing neurons were located within L2/3 (Supplementary Fig. 6), supporting the conclusion that the differences reflected connectivity between L2/3 and L5 neurons.

**Fig. 7:**
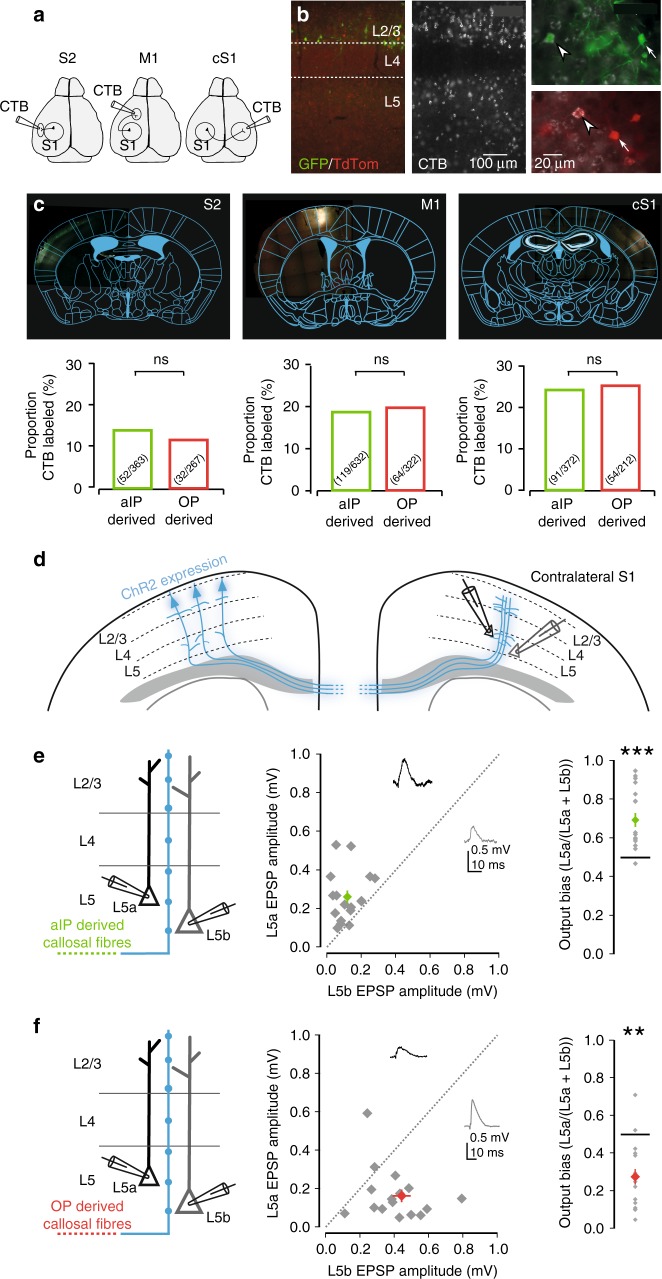
Long-range fine-scale synaptic targeting by aIP-derived L2/3 neurons. **a** Mice that had undergone IUE reached adulthood and received an injection of the retrograde tracer, Choleratoxin β-subunit (CTB), into either secondary somatosensory cortex (S2), primary motor cortex (M1), or primary somatosensory cortex of the hemisphere contralateral to that which had been electroporated (cS1). **b** Immunofluorescence staining of S1 was used to determine cortical layers and the proportion of aIP- and OP-derived neurons retrogradely labeled with CTB (left, middle). Example images (right) show TdTomato^+^ and GFP^+^ L2/3 neurons that either did (arrowheads), or did not (arrows), show retrograde labeling. **c** Coronal sections (top) show representative CTB injections in S2, M1 or cS1. Bar plots (bottom) show that similar proportions of aIP- and OP-derived L2/3 neurons project to each region. **d** Optogenetic experimental design for studying cell-type specific targeting by long-range callosal projections. Simultaneous recordings from pairs of L5a and L5b pyramidal neurons were used to assess fine-scale targeting from L2/3 to cS1. **e** aIP-derived L2/3 pyramidal neurons preferentially drove contralateral L5a, compared with L5b (response bias toward contralateral L5a of 0.68 ± 0.04, *p* = 0.0002, Wilcoxon signed rank test, *n* = 17 pairs). **f** OP-derived L2/3 pyramidal neurons tended to provide stronger input to contralateral L5b pyramidal neurons (response bias toward contralateral L5b of 0.28 ± 0.04, *p* = 0.001, Wilcoxon signed rank test, *n* = 17 pairs). Error bars represent standard error of the mean. Source data are provided as a Source Data file

In addition to translaminar connections within the same column, L2/3 pyramidal neurons within S1 are known to send synaptic outputs to other cortical regions, in a manner that is typically non-overlapping and thought to be important for sensorimotor integration^45–48^. We used localized injections of the retrograde label, choleratoxin β-subunit (CTB), to examine whether aIP- and OP-derived L2/3 neurons differ in terms of their long-range projections from S1 to secondary somatosensory cortex (S2), primary motor cortex (M1), and contralateral S1 (cS1; Fig. 7a, b). Quantitative anatomical analysis revealed that there was no significant difference in the proportion of aIP- and OP-derived L2/3 neurons that projected to S2 (*p* = 0.41, Fisher’s exact test, *n* = 267 and 363 neurons from four animals), to M1 (*p* = 0.73, Fisher’s exact test, *n* = 322 and 632 neurons from four animals) or to cS1 (*p* = 0.84, Fisher’s exact test, *n* = 212 and 372 neurons from three animals; Fig. 7c).

While we did not detect differences in the probability of projecting to a cortical region, we considered that the aIP-derived L2/3 population might still exhibit a relative bias to target L5a post-synaptic neurons, similar to that observed for ipsilateral translaminar connections from L2/3 to L5 (Fig. 6). To test this, we focused on the callosal projections from L2/3 pyramidal neurons in S1, to L5 neurons in cS1. Consistent with previous work^49^, our retrograde labeling confirmed that the callosally-projecting aIP- and OP-derived labeled neurons were almost exclusively located in L2/3 (Supplementary Fig. 7). Thus, an optogenetic strategy could be used to map interhemispheric synaptic inputs from L2/3 to L5, by selectively expressing ChR2 in either aIP- or OP-derived neurons of one hemisphere, and activating their callosal axons in cS1. Simultaneous whole-cell patch-clamp recordings were made from pairs of L5a and L5b pyramidal neurons in cS1 (Fig. 7d). Consistent with previous studies, ChR2-expressing callosal axons were relatively sparse in the contralateral cortex and photostimulation elicited responses that were smaller than in ipsilateral cortex^50^. However, upon stimulating callosal axons, we saw a similar connectivity profile to that observed in the ipsilateral hemisphere. Activating aIP-derived callosal axons resulted in a strong tendency to drive the L5a pyramidal neuron over the L5b neuron, with an output bias toward L5a of 0.68 ± 0.04, where a value of 0.5 represents equal output (*p* = 0.0002, Wilcoxon signed rank test, *n* = 17 pairs; Fig. 7e). In contrast, when callosal axons from OP-derived neurons were activated, we observed a pronounced bias in the opposite direction, such that OP-derived L2/3 neurons preferentially drove the contralateral L5b pyramidal neuron over the L5a neuron (bias toward L5b of 0.28 ± 0.04, *p* = 0.001, Wilcoxon signed rank test, *n* = 17 pairs; Fig. 7f). Together, these data support the conclusion that a glutamatergic neuron’s progenitor type of origin is associated with both local and long-range fine-scale post-synaptic targeting, and implicates aIPs in the generation of diverse excitatory subnetworks.

## Discussion

A combination of transcriptomic, morphological and functional single-cell methods was used to examine the contribution of embryonic progenitor pools to cortical diversity in the mouse. Excitatory neuronal populations derived from apical intermediate progenitors (aIP-derived) were compared with layer-matched neurons labeled at the same stage of embryonic development, but derived from other pools of progenitors (OP-derived). Targeted single-cell RNA sequencing revealed that the different progenitors contribute to common populations of transcriptionally defined L4 and L2/3 glutamatergic cell types, but that the output of aIPs is biased and more restricted when compared with that of OPs. The neurons showed similar layer-related morphologies and intrinsic electrical properties, whereas assessment of post-synaptic target selection proved very effective in discriminating the neuronal populations. aIP-derived neurons avoid local intralaminar monosynaptic connections with one another, preferring to connect to neighboring neurons derived from OPs. Furthermore, the post-synaptic targeting of extralaminar projections to deeper layers of cortex also reflects the progenitor pool of origin. aIP-derived neurons preferentially target L5a, which directly relays information to other regions of cortex. aIPs therefore generate diversity at the level of fine-scale cortical subnetworks that differentially route excitatory synaptic information.

Our observations support the general view that excitatory cortical neurons are derived from a heterogeneous population of progenitors located in the embryonic ventricular and subventricular proliferative zones^4,20,23,26,28,30–35,51,52^. Our single-cell RNA-sequencing studies provide the first evidence on how neural progenitor pools give rise to distinct subsets of cortical glutamatergic neurons. Using a recently published large-scale cell classification scheme based on single-cell RNA sequencing in mouse cortex^3^, simultaneously proliferating progenitor pools are shown to differ in their contribution to the cellular composition of a cortical layer. While both aIPs and OPs generate the *Rspo1* glutamatergic neurons within L4, the contribution of aIPs to the population of L2/3 glutamatergic neurons was biased and more restricted when compared with that of OPs. aIPs almost exclusively generated *Agmat* L2/3 pyramidal neurons, whereas OPs generated a mixture of *Agmat*, *Adamts2*, and *Rrad* L2/3 glutamatergic neurons.

The biased output of transcriptomically classified neurons from aIPs supports the idea that intermediate progenitors have emerged to increase the representation of particular post-mitotic cell types^26,29,35,53^, and also supports the idea that pallial VZ neuronal progenitors can exhibit different degrees of lineage restriction^23,51,52^. At the same time, as aIPs are derived from radial glial cells, our findings are compatible with a general model in which a single neuronal progenitor cell type ultimately gives rise to the full complement of excitatory cortical neuronal cell types^24,29^. Within this model our data argue that multiple excitatory progenitor pools, and intermediate progenitor pools in particular, have not simply evolved to expand cortical volume, but also contribute to cortical diversity.

Fine-scale connectivity and the selection of post-synaptic partners proved a particularly sensitive discriminator of aIP-derived neurons from neighboring OP-derived progeny^54^. Our observations therefore advance the idea that the selection of post-synaptic partners is closely linked to a neuron’s lineage^16–19,55^. Previous work has shown that the probability of certain translaminar excitatory monosynaptic connections is higher between two cortical neurons derived from the same individual progenitor; that is, between two ‘sister’ neurons derived from the same ‘mother’ progenitor cell^18^. The current study establishes that a key factor underlying a cortical neuron’s selection of both intralaminar and extralaminar post-synaptic partners can be the embryonic progenitor type from which the neuron is derived. This has broad implications for understanding the formation of cortical connectivity because a cortical neuron forms synapses with many hundreds of other excitatory neurons^1,38^, the vast majority of which will be derived from different individual progenitors that can be classified into particular types.

Simultaneous patch-clamp recordings revealed that within both L4 and L2/3 of the cortex, neurons derived from the aIP pool show a strong tendency to form local monosynaptic intralaminar connections with neighboring neurons derived from OPs. In L4, the pattern resulted from a lower probability of connection between aIP-derived neurons. While in L2/3, the pattern resulted from a higher level of connectivity from aIP-derived neurons to OP-derived neurons. This represents the first evidence that lateral excitatory connectivity reflects a cortical neuron’s progenitor type, and could underlie previous observations that intralaminar connectivity can be associated with axo-dendritic overlap, similarity in sensory response properties and the sharing of common long-range inputs or outputs^9–11,13–15,56^.

Examination of extralaminar cortical connectivity provided further evidence that post-synaptic target selection can reflect embryonic progenitor type. As previously reported for the L2/3 to L5 translaminar connection, aIP-derived L2/3 neurons generated post-synaptic responses in neurons whose soma were located throughout L5^38^. At the population level, however, aIP-derived L2/3 neurons provided more excitatory drive to L5a than to L5b neurons, while the opposite was true for OP-derived L2/3 neurons. Indeed, the same bias was also evident in the fine-scale connections made with L5 neurons in the contralateral hemisphere, suggesting that this emerges through local interactions between the pre-synaptic axons and post-synaptic dendrites.

As post-synaptic partner selection results from an interplay of molecular and activity-dependent processes, one could imagine multiple mechanisms that might contribute to the link between synaptic connectivity and progenitor pool. In terms of cell type differences, the progenitor-associated synaptic connectivity observed in L2/3 could reflect the properties of particular cell populations. For instance, one could imagine that *Adamts2* and *Rrad* L2/3 neurons show a strong bias to project to L5b and a high probability of receiving intralaminar inputs from *Agmat* neurons. However, the fact that aIP-derived L4 neurons show a similar out-of-class connectivity rule suggests that mechanisms beyond cell-type also contribute. Indeed, it has been shown that cortical neurons can inherit particular molecular and/or activity-dependent identities from their progenitors, which influence synaptic connectivity. For example, progenitor-related differences in gap junction coupling can influence activity-dependent mechanisms of synapse selection^17^, and progenitor-related differences in the epigenetic regulation of cell-adhesion molecules has also been implicated in synaptic partner selection^56^.

The in utero labelling strategy used here distinguished two progenitor pools at a defined embryonic stage and tracked their progeny into the mature brain. Although the method allowed us to distinguish neurons derived from different progenitor pools, it provided limited information on the lineage pathways taken by the neurons. Previous work has suggested that aIP divisions tend to produce post-mitotic neurons directly, rather than via other progenitors^22^. Indeed, we replicated the observation that aIP-derived L2/3 neurons resided somewhat further from the pial surface than OP-derived L2/3 neurons, supporting the idea that they have different lineage pathways^22^. However, how aIPs relate to other progenitor types, and whether there are also subtypes of aIPs, requires further investigation. Similarly, the labelling method did not distinguish between OP-derived neurons that may have followed different lineage routes. Radial glial cells within the OP population are known to undergo self-renewing cell-divisions that either generate neurons directly or via intermediate progenitor cells, including outer radial glia and basal intermediate progenitor cells within the SVZ^20,24,28,29,32–34^. Given this heterogeneity, it remains unknown how these different progenitor types contribute to the neuronal differences observed here. For these reasons, it will be informative to develop strategies for further isolating defined progenitor subpopulations, while also tracking their neuronal progeny through different lineage pathways to maturity.

Another outstanding question is how the birthdate of neurons (i.e., time since becoming post-mitotic) contributes to the neuronal differences that we observed. Within the OP-derived or aIP-derived populations we observed different cell types across cortical layers, which is clearly consistent with a contribution of birthdate. It is also possible that the neuronal differences observed within a layer could reflect differences in precise birthdates^57^. Indeed, birthdate differences may be linked to progenitor types. Although the within-layer differences in cell-type and synaptic connectivity were observed for neighboring neurons that were matched in terms of their distance to the pial surface. Hence any age-effects would have to operate beyond the somatic position within cortex, perhaps as a result of different migration patterns used by cohorts of neurons.

Progenitor pools that exhibit different potentials may reflect limitations in terms of the cell types or numbers that can be generated by an individual progenitor. By linking progenitor pool to the synaptic connectivity of neurons, our observations support the idea that progenitors with different potential may also have evolved to generate specific patterns of excitatory cortical connectivity. Indeed, the fact that progenitor type predicts both the intralaminar and extralaminar connectivity of upper layer neurons, suggests that different progenitor populations give rise to excitatory cortical subnetworks that are defined by specific arrangements of local and long-range connections^9,13,14^. Such subnetworks provide a substrate for neighboring neurons to process information independently^12,13^ or to integrate multiple streams of sensory and/or motor-related information^10,11,14^. Indeed, our description of aIP-derived neurons could account for an earlier observation that non-connected L2/3 pyramidal neurons are more likely to converge onto the same L5 pyramidal neurons in somatosensory cortex^14^. L5 is a key stage in the routing of excitatory synaptic information through cortex, with L5b mediating transmission to subcortical targets and L5a representing a direct route for transmitting information between cortical areas^40^. Therefore, via both their local connectivity and outputs to deeper layers, aIP-derived subnetworks are well-placed to share excitatory information that could derive from different sources or levels of cortical processing. More generally, these observations raise the possibility that through their progenitor composition, cortical areas could acquire different combinations of excitatory subnetworks for performing particular types of computation.

## Methods

### Animals

All experiments were carried out on C57BL/6 wild-type mice, which were bred, housed and used in accordance with the UK Animals (Scientific Procedures) Act (1986). Females were checked for plugs daily and the day of the plug was considered embryonic day (E) 0.5.

### In utero electroporation

In utero electroporation (IUE) was performed using standard procedures. In short, pregnant females were anaesthetized using isoflurane and their uterine horns were exposed by midline laparotomy. A mixture of plasmid DNA (1.5 μg/μl) and 0.03% fast green dye was injected intraventricularly using pulled micropipettes through the uterine wall and amniotic sac. Plasmid DNA included: (i) ‘Tα1-Cre’, in which the gene for Cre recombinase is under the control of a portion of the *Tα1* promoter^22^; (ii) ‘CβA-FLEx’, which uses the chicken β-actin promoter to control a flexible excision (FLEx) cassette, whereby Cre recombination permanently switches expression from TdTomato fluorescent protein to enhanced green fluorescent protein (GFP)^23^; (iii) ‘DIO-ChR2-mCherry’ (pAAV-EF1a-doublefloxed-hChR2(H134R)-mCherry-WPRE-HGHpA; Addgene #20297), in which Cre recombination permanently turns on the expression of channelrhodopsin-2 (ChR2) under the control of the human elongation factor-1a promoter^44^; and (iv) DO-ChR2-mCherry (‘Cre-Off’; pAAV-Ef1a-DO-hChR2(H134R)-mCherry-WPRE-pA; Addgene #37082 in which Cre recombination permanently turns off the expression of ChR2 under the control of the human elongation factor-1a promoter^44^. Total volume injected per pup was ~1 μl. Tα1-Cre and CβA-FLEx plasmids were injected as a 1:1 ratio of plasmid DNA (each 3 μg/μl, so that the final concentration of each plasmid was 1.5 μg/μl). The anode of a Tweezertrode (Genetronics) was placed over the dorsal telencephalon outside the uterine muscle. Five pulses (50 ms duration separated by 950 ms) at 42 V (for E14.5 and E15.5), 40 V (for E13.5) and 38 V (for E12.5) were delivered with a BTX ECM 830 pulse generator (Genetronics). The uterine horns were placed back inside the abdomen, the cavity filled with warm physiological saline and the abdominal muscle and skin incisions were closed with vicryl and prolene sutures, respectively. Dams were placed in a clean cage and monitored until the birth of the pups.

### Postnatal injections

Animals that had undergone IUE of Tα1-Cre and CβA-FLEx plasmids were used for targeted intracerebral injection of the retrograde tracer Choleratoxin subunit B (CTB) (6–12 weeks postnatal). Briefly, postnatal mice were deeply anaesthetized using isoflurane and placed in a stereotaxic frame (Kopf Instruments). Buprenorphine (0.1 mg/kg) was administered subcutaneously, and EMLA cream was applied to the scalp. An incision was made to expose the skull, bregma and lamda were located and then a small craniotomy was performed to expose the neocortex. Injections were made with a pulled glass micropipette (Blaubrand intraMARK). Intracerebral injections involved delivering 1% CTB (Sigma) with 0.03% Fast Green at a rate of 33 nl/min into either ipsilateral S2 (0.6 mm posterior, 4.7 mm lateral, 66 nl injected at 500 μm deep), ipsilateral M1 (1 mm anterior, 1.3 mm lateral, 132 and 66 nl injected at depths of 700 and 300 μm deep from pia) or contralateral S1 (1.2 posterior 2.5 mm lateral 132 and 66 nl injected at depths of 700 and 300 μm). The injection pipette was maintained in place for an additional 5 min before slow withdrawal. The craniotomy was covered; the skin wound closed with vicryl sutures and the animal was recovered in a heated chamber.

### Slice preparation and recording conditions

Acute cortical slices were generated from postnatal animals for single-cell transcriptomics studies (at either P10 or P30) and electrophysiological recordings (P21–P35). Animals were anaesthetized with isoflurane and then decapitated. Coronal 350–400 µm slices were cut using a vibrating microtome (Microm HM650V). Slices were prepared in artificial cerebrospinal fluid (aCSF) containing (in mM): 65 Sucrose, 85 NaCl, 2.5 KCl, 1.25 NaH_2_PO_4_, 7 MgCl_2_, 0.5 CaCl_2_, 25 NaHCO_3_ and 10 glucose, pH 7.2–7.4, bubbled with carbogen gas (95% O_2_/5% CO_2_). Slices were immediately transferred to a storage chamber containing aCSF (in mM): 130 NaCl, 3.5 KCl, 1.2 NaH_2_PO_4_, 2 MgCl_2_, 2 CaCl_2_, 24 NaHCO_3_ and 10 glucose, pH 7.2–7.4, at 32 °C and bubbled with carbogen gas.

When required, slices were transferred to a recording chamber and continuously superfused with aCSF bubbled with carbogen gas with the same composition as the storage solution (32 °C and perfusion speed of 2 ml/min). Whole-cell current-clamp recordings were performed using glass pipettes, pulled from standard wall borosilicate glass capillaries and containing (in mM): 110 potassium gluconate, 40 HEPES, 2 ATP-Mg, 0.3 Na-GTP, 4 NaCl and 4 mg/ml biocytin (pH 7.2–7.3; osmolarity, 290–300 mosmol/l). Neurons that were >40 μm below the surface of the slice were targeted and recordings were made using Multiclamp 700 A, Multiclamp 700B and Axoclamp 2B amplifiers and acquired using pClamp9 (Molecular Devices, RRID:SCR_011323) or WinWCP software (University of Strathclyde, UK, RRID:SCR_014713).

### Single-cell collection and RNA sequencing

Individual neurons were collected and analyzed using an adapted version of the Patch-seq method^36,37^, optimized for speed, such that a minimum of 16 neurons could be collected from each mouse. Neighboring TdTomato^+^ and GFP^+^ neurons were collected alternately from the same cortical layer of region S1 in acutely prepared brain slices. Batches of neurons were then submitted for RNA sequencing in 96-well plate format, which comprised a single age (P10 or P30) and cortical layer (L2/3 or L4). For neuron collection, thin-walled borosilicate glass capillaries (1.2 mm outer diameter; Harvard Apparatus) were pulled using a Flaming Brown micropipette puller (Sutter Instrument Company) to obtain an aperture of ~5 μm. The micropipette was filled with 2.4 μl lysis buffer (0.2% v/v Triton X-100 (Sigma-Aldrich) containing 2 U/μg RNase inhibitor (Takara)^58^. Fluorescently labeled cells were approached with positive pressure and, after releasing the pressure, contact was made with the cell membrane and negative pressure was applied to achieve the cell-attached configuration (a ‘gigaseal’). The membrane was then ruptured and the cell contents were aspirated into the micropipette with light suction sustained over ~1 min. The whole process was visualized under fluorescence and brightfield illumination in order to confirm entry of the cell into the pipette. Pressure was equilibrated and then fixed as the pipette was withdrawn from the perfusion chamber. The contents of the micropipette were transferred into a 0.2 ml PCR tube (4titude), by application of strong positive pressure once the tip was located near the bottom of the PCR tube. To ensure the cell was transferred, the tip of the micropipette was finally broken off on the side of the PCR tube. Samples were stored on dry ice for up to 4 h and transferred to −80 °C until processing. Tubes were inserted into a rigid, fully skirted frame for 96 samples and the location of each cell in the plate was randomized and recorded. Samples of cell lysis buffer and 10 pg human RNA served as negative and positive controls, respectively.

Single cell RNA-seq was performed at the Oxford Genomics Centre, University of Oxford. Sequencing libraries were prepared following the Smart-seq2 protocol^58^, adding 1 μl ERCC spike-in (Thermo Fisher; ref. ^59^). The prepared libraries were combined into a 96-plex pool and sequenced over one lane of HiSeq 4000 (Illumina) with paired-end 75 bp reads. Trimmomatic v. 0.32^60^ was first used to discard low-quality reads, trim adaptor sequences, and eliminate poor-quality bases using the options LEADING:3 TRAILING:3 SLIDINGWINDOW:4:15 MINLEN:36. Reads were mapped to a modified GRCm38 (mm10) mouse genome (Genome Reference Consortium, 2011), which included 92 ERCC spike-ins and TdTomato and GFP sequences of the CßA-FLEx construct using the *STAR* aligner (v2.4.2a^61^; with ‘--outFilterMismatchNmax 999 --outFilterMismatchNoverLmax 0.05’ options to set a maximum number of mismatches that is proportional to read length. To quantify gene expression levels, *featureCounts*^62^ was used to count the number of reads that mapped uniquely to annotated exon features using a modified gene annotation file, which included Ensembl GRCm38.88 gene annotations, 92 ERCC spike-ins, TdTomato and GFP annotations, and the ‘-Q 10 -p -B’ options, which impose a minimum mapping quality score and more stringent requirements for paired-end alignment and counting.

### Single-cell RNA-seq analysis and cell classification

Low-quality cells were filtered using *scater* (v1.10.0^63^; based on library size and number of expressed genes using a cutoff of >3 median absolute deviations (MADs) below the log transformed median. A cutoff of 5 MADs above the median was employed for the proportion of mitochondrial genes, and 4 MADs above the median was used for the proportion of reads mapping to ERCCs. Histograms of the number of reads, number of genes expressed and the proportion of mitochondrial genes were plotted following filtering (Supplementary Fig. 4a-c). Data was initially normalized using *scran* (v.1.10.1; ref. ^64^). ERCCs were not used for normalization. Even after *scran* normalization, principal component 1 strongly correlated with the number of genes expressed. Further normalization was therefore performed using the RUVs method from *RUVSeq* (v1.16.0; ref. ^65^) using the option *k* = 1 (Supplementary Fig. 4d). External datasets were obtained and normalized using *scran* and normalized counts were merged using the mnnCorrect method from *scran*.

For cell classification, *scrattch.hicat* (https://github.com/AllenInstitute/hicat) was used on log2 normalized counts per million (cpm) data. The classifier utilized the unique median expression profile of each cluster (i.e., class) of cortical cells derived from the recent Allen Brain Institute dataset^3^, restricting our analysis to all cell classes represented in the visual cortex (115 cell types, excluding ‘low quality’ classes according to the original annotation). Marker genes for the expression profiles were selected based on differential expression between cell classes in the reference dataset. Each aIP- and OP-derived cell was then mapped to the class with the highest correlation value, using 80% of marker genes randomly over 100 bootstrapping iterations to provide a measure of certainty. To be classified, cells had to exhibit more than 50% certainty of being correlated to a single-cell class. Any cell that did not meet this criteria or was correlated to one of the ‘low quality’ classes, was deemed as ‘unclassified’^3^.

An investigation of differentially expressed genes between the aIP- and OP-derived populations recovered the fluorescent markers as expected, but did not identify statistically significant differential expression of endogenous genes that were robust to permutation testing. Further examination using gene set enrichment analysis (GSEA) also did not reveal the enrichment of gene categories outside the constraints of a pre-determined *p*-value. To perform the differential expression analysis, counts data obtained from *featurecounts* was transformed using *zinbwave* (v1.4.0; ref. ^66^) to provide a low-dimensional representation of zero-inflated negative binomial reads. Differential expression analysis was subsequently performed using *DESeq2* (v1.22.1; ref. ^67^). Library complexity bias was corrected using the W_1 factor obtained from *RUVSeq* and inserted into the model ~group+W_1. Permutation analysis was performed with replacement. Gene set enrichment analysis (GSEA; ref. ^68^) was carried out using the *fgsea* implementation (v1.8.0; ref. ^69^). Genes were pre-ranked on log2 fold change, placing the strongest upregulated and downregulated genes at the top and bottom of the list, respectively. In total, 10,000 gene set permutations were used for the analysis, and clusters smaller than 15 and larger than 500 genes were discarded. Gene sets were selected from the GSKB GO gene sets (~4300 gene sets; ref. ^70^). Custom code has been made available at https://github.com/jscaber/cgat-proj057.

### Electrophysiological stimulation and recording protocols

To assess intralaminar connectivity, we performed quadruplet recordings comprising two aIP-derived and two OP-derived neurons within the same cortical layer, but accepted triplet or paired recordings if we were unable to establish a quadruplet. The data were therefore derived from a mixture of quadruplet (*n* = 180 neurons), triplet (*n* = 171 neurons) and paired (*n* = 190 neurons) recordings. For the optogenetic studies, all data were derived from paired recordings. Assessment of intralaminar connectivity was performed by delivering brief (~5 ms) suprathreshold current injections (1 nA) or brief trains of current injections (6 pulses, 5 ms, 1 nA at 20 Hz) to each patched neuron sequentially, while simultaneously recording the membrane voltage of the other neurons. This was repeated 10–20 times. Synaptic delay was the time from the pre-synaptic action potential to when the post-synaptic response reached 20% of its maximum. A variety of protocols consisting of hyperpolarizing and depolarizing current steps were used to assess the intrinsic properties of the recorded neurons, including input resistance, spike threshold (10 pA incremental current steps) and action potential frequency (current steps ranging from −300 to +600 pA, 100 pA steps). The ‘out-of-class’ connectivity bias was defined as the probability of connecting to neurons derived from a different progenitor pool, divided by the sum of probabilities of connecting to neurons derived from all progenitor pools.

Photoactivation of ChR2 was achieved using 1–2 ms duration light pulses via a diode-pumped solid-state laser (473 nm peak wavelength; Shanghai Laser and Optics Century). The laser was coupled to a 200 μm diameter multimode optic fiber via a collimating lens (Thorlabs). The tip of the optic fiber was positioned at an image plane in the microscope in the center of the optical axis and directed into a 20X/1.0 numerical aperture objective lens via a dichroic mirror. Illumination at the slice (0.3–4.2 mW mm^−2^) was controlled by adjusting the laser power to generate low amplitude monosynaptic EPSPs (<4 mV), so as to minimize the recruitment of polysynaptic activity.

The output from L2/3 pyramidal neurons to L5 pyramidal neurons was studied through selective optical activation of axons from either aIP- or OP-derived neurons using ChR2-mCherry. IUE for these experiments was performed at E14.5 or E15.5. The EPSP latencies following optical activation were consistent with monosynaptic responses and were similar for ipsilateral EPSPs from aIP-derived neurons (3.89 ± 0.42 ms and 4.38 ±  0.54 ms from laser onset for L5a and L5b, respectively) and OP-derived neurons (3.71 ± 0.36 ms and 4.32 ± 0.52 ms from laser onset for L5a and L5b, respectively; *p* > 0.05 in all cases, *t*-test). Latencies were also similar for contralateral EPSPs from aIP-derived neurons (3.51 ± 0.59 ms and 4.38 ± 0.66 ms from laser onset for L5a and L5b, respectively) and OP-derived neurons (4.76 ± 1.10 ms and 3.08 ± 0.62 ms for L5a and L5b, respectively; *p* > 0.05 in all cases, *t*-test).

### Analysis of electrophysiological recordings

Data were analyzed offline using custom written programs in Igor Pro (Wavemetrics, RRID:SCR_000325). Synaptic connectivity was assessed by averaging the 10–20 sweeps of single spike or trains of spike stimulation and detecting excitatory post-synaptic potentials (EPSPs). These were defined as upward deflections of more than 2 standard deviations (SD) above baseline. The input resistance was calculated by dividing the membrane potential observed after hyperpolarizing the membrane potential with −300 pA current. The analysis of EPSP kinetics (peak amplitude, duration, rise time, and decay time) was performed on average synaptic responses. Analysis of optically evoked excitatory synaptic input to L5 neurons was performed by averaging 10–30 sweeps in which the pre-synaptic ChR2 fibers were activated.

### Histological analyses

Following whole-cell patch-clamp recording, acute brain slices were fixed in 4% paraformaldehyde in 0.1 M phosphate buffer (PB; pH 7.4). Biocytin-filled cells were visualized using streptavidin fluorescent-conjugated antibodies and DAB immunohistochemistry was performed using standard procedures. To delineate cortical layers, slices were co-stained with the nuclear marker 4′,6-diamidino-2-phenylindole (DAPI) in PBS (1:100,000). For whole-brain histology, brains were either fixed by cardiac perfusion of phosphate buffered saline (PBS) followed by 4% paraformaldehyde in 0.1 M phosphate buffer (PFA; pH 7.4), or by rapid decapitation of the head and submersion in oxygenated sucrose cutting solution before submersion in 4% PFA. Brains were stored in 4% PFA for an additional 24–72 h, after which they were washed in PBS. Whole-brain tissue was sectioned at 50–60 μm on a vibrating microtome (VT1000S; Leica Microsystems).

The standard immunohistochemistry protocol was as follows. Sections were washed three times in PBS for 5 min and permeabilized in PBS-Triton-X (0.1/0.3% for embryonic and adult tissue, respectively; PBST) for 30 min. Subsequently, sections were blocked in 10% normal serum (Vector labs) in PBS for at least 1 h at RT, washed with PBS and incubated overnight at 4 °C in primary antibody diluted in PBST (0.1/0.3%) and 1–10% serum. Primary antibodies included anti-pH3 (1:500, rabbit; Millipore, CAT# 06-570, RRID:AB_310177), anti-Ki-67 (1:500, rabbit; Abcam, CAT# ab15580, RRID:AB_443209), anti-VGLUT2 (1:250, rabbit; Synaptic Systems, CAT# 135 403, RRID:AB_887883), anti-RFP (1:500, rat; Chromotek, CAT# 5f8-100, RRID:AB_2336064), anti-GFP (1:1000, chicken; Aves Lab, CAT# GFP-1020, RRID:AB_10000240) and anti-beta subunit choleratoxin (1:500, mouse Abcam, CAT# ab35988, RRID:AB_726860). VGLUT2 staining was facilitated through heated antigen retrieval at 80 °C in 10 mM sodium citrate buffer (pH 6.0) for 30 min prior to incubation. Slices subsequently received three 10 min washes in PBS and were then incubated overnight at 4 °C, or for 2 h at RT, with secondary antibodies diluted in 0.3% PBS-Triton-X. Secondary antibodies included anti-rat alexa fluor 568 (1:500, goat; Thermo Fisher Scientific, CAT# A-11077, RRID:AB_2534121), anti-rabbit-Cy5 fluorophore (1:500, donkey; Jackson Laboratories, CAT# 711-175-152, RRID:AB_2340607) and anti-mouse alexa fluor 680 (1:500, goat; LiCor Technologies, CAT# 925-68070, RRID:AB_2651128). Sections were counter-stained with DAPI (1:10,000) and mounted on slides with VectaShield (Vector labs, CAT# H-1000, RRID:AB_2336789). For the labeling of basal processes in progenitor cells, a solution of the lipophilic dye 1,1′-dioctadecyl-3,3,3′,3′-tetramethylindotricarbocyanine iodide DiR (1 mg/ml in DMSO, Thermo Fisher Scientific, CAT# D12731) was applied directly to the dorsal surface of fixed E15.5 brains using a paintbrush. Brains were stored in 4% PFA at RT for 4–6 weeks to allow for labeling of VZ cells with basal processes, were then sectioned and incubated for a 2 min in DAPI, after which they were immediately imaged. Fluorescence images were captured with a LSM 710 confocal microscope using ZEN software (Zeiss, RRID:SCR_013672) or Leica DM5000B epifluorescence microscope using Openlab software (PerkinElmer, RRID:SCR_012158). DAB-immunoreactive neurons were reconstructed using Neurolucida and Neuroexplorer software (MBF Bioscience, Williston, USA, RRID:SCR_001775).

Cell counting and localization was performed using ImageJ software (RRID:SCR_003070). Positive cells had a fluorescence signal that was at least twice the background fluorescence (measured from randomly selected regions of the tissue). *x*- and *y*-coordinates of aIP- and OP-derived neurons were used to calculate the distance from pia and lateral spread in L2/3 and L4. Counting of progenitor cell basal processes was performed in *z*-stack projections of confocal stacks of ~40 μm thickness. All clearly delineated processes above the SVZ and extending to the pial surface were counted.

### Statistics

All data are presented as means ± SEM. Statistical tests were all two-tailed and performed in SPSS 17.0 (IBM SPSS statistics, RRID:SCR_002865), GraphPad Prism version 5.0 (GraphPad Software, RRID:SCR_002798) or MATLAB (RRID:SCR_001622). Contingency tables were assessed with the Fisher’s exact test. Continuous data were assessed for normality and appropriate parametric (paired *t*-test, unpaired *t*-test) or non-parametric (Wilcoxon signed rank, Mann–Whitney U, Kruskal–Wallis) statistical tests were applied (*p < 0.05, **p < 0.01, ***p < 0.001).

### Reporting summary

Further information on research design is available in the Nature Research Reporting Summary linked to this article.

## Supplementary information

Supplementary Information

Peer Review

Reporting Summary

Source Data file

## Data Availability

All data are available upon reasonable request. The source data underlying Figs. 1e–f, 2b–g, 3a–d, 4d, e, 5d, e, 6d–f, 7c-f, Supplementary Figs 1d-e, 2c, 3b-g, 4a-d, 5a-b, 6b-f, 7b-c, and Supplementary Tables 1 and 2 are provided as a Source Data file.

## Code availability

Custom code is publicly available and can be accessed via GitHub (https://github.com/jscaber/cgat-proj057).
