# Supplementary material for: Embryonic progenitor pools generate diversity in fine-scale excitatory cortical subnetworks
Source: Nat Commun. 2019 Nov 19;10:5224. doi: 10.1038/s41467-019-13206-1 (PMC6863870; doi:10.1038/s41467-019-13206-1)
Supplement: Supplementary file 1 — Supplementary Information [file 41467_2019_13206_MOESM1_ESM.pdf]

Supplementary Materials for

Title: Embryonic progenitor pools generate diversity in fine-scale excitatory cortical subnetworks

Ellender et al.

This PDF file includes:

Supplementary Figures 1 to 7
Supplementary Tables 1 and 2

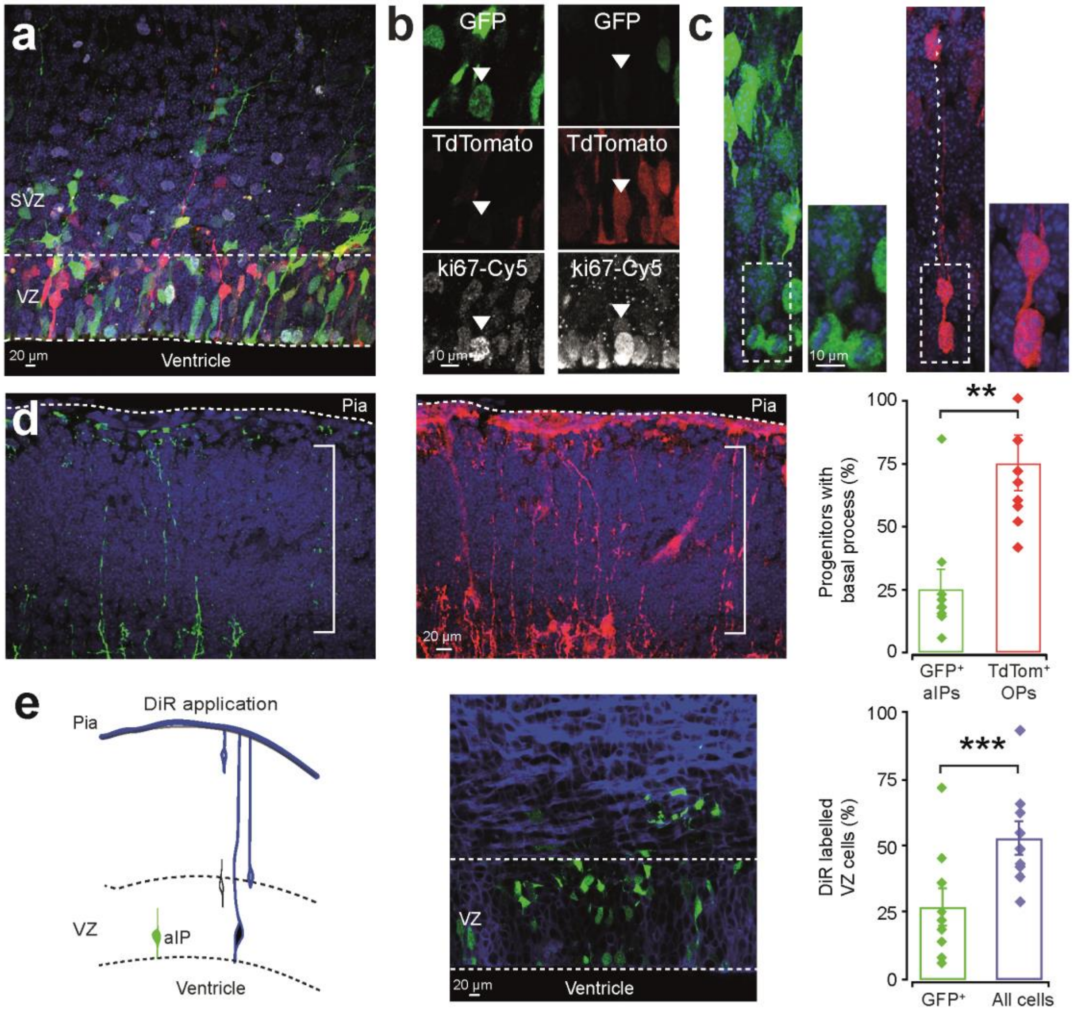

Supplementary Figure 1 ***In utero electroporation*** **of Tα1-Cre and CβA-FLEx plasmids labels distinct progenitor pools.**

(**a**) Tα1-Cre and CβA-FLEx plasmids were delivered by IUE at E14.5. 24 h later, confocal imaging revealed GFP^+ progenitors that corresponded to apical intermediate progenitors (aIPs; also referred to as short neural precursors) and $TdTomato^+$ (GFP^-) progenitors that corresponded to other progenitors (OPs). (**b**) Both progenitor pools were actively dividing in the VZ, as determined by the expression of Ki-67. (**c**) During mitosis at the ventricular wall, GFP^+ aIPs lacked a basal process (left), whilst $TdTomato^+$ OPs retained a basal process (right; dashed line). (**d**) Projection of confocal stack of GFP^+ (left) and $TdTomato^+$ (middle) processes in the embryonic cortex, 24 h after IUE. Whilst the majority of $TdTomato^+$ OPs had a basal process that extended through the SVZ to the pial surface, this was the case in a minority of GFP^+ aIPs (right) ($TdTomato^+$: 75.5 +/- 11.0 %, GFP^+: 25.4 +/- 7.8 %; p = 0.007, t-test, n = 9). (**e**) Application of the lipophilic dye, DiR, to the pial surface of E15.5 brains (left) labeled VZ cells via their basal process (middle). Compared to all DAPI-stained cells in the VZ, GFP^+ aIPs showed significantly less DiR labeling, consistent with shorter basal processes (All VZ cells: 52.7 +/- 6.3 % and GFP^+ only cells: 27.1 +/- 7.0 %; p = 0.0004, t-test, n = 9).). Error bars represent standard error of the mean. Source data are provided as a Source Data file.

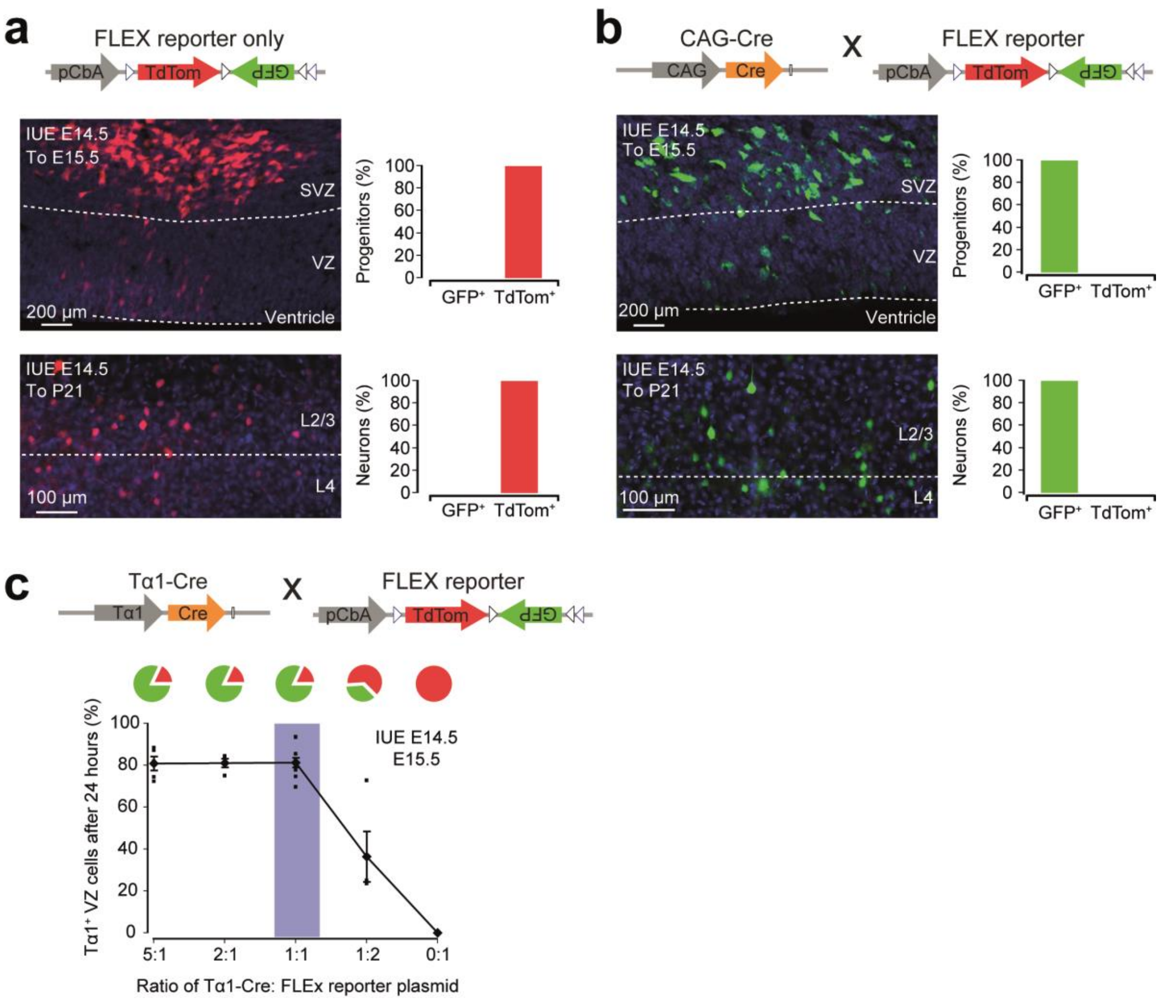

Supplementary Figure 2 Reporter plasmid faithfully reports cre recombinase expression.

(**a**) IUE at E14.5 of the CβA-FLEx reporter plasmid alone resulted in only TdTomato^{+} progenitor cells at 24 h (top; n = 4) and only TdTomato^{+} cortical neurons at P21 (bottom; n = 3). This excludes the possibility of spontaneous recombination of the CβA-FLEx reporter. (**b**) IUE at E14.5 of the CβA-FLEx reporter plasmid and a second plasmid, in which a ubiquitous promoter (‘CAG-Cre’) drove Cre recombinase, resulted in complete and rapid recombination in all progenitors within 24 h (top; n = 3), which was maintained in cortical neurons at P21 (bottom; n = 2). (**c**) Quantification of GFP^{+} and TdTomato^{+} cells in the VZ, 24 h after IUE with different ratios of Tα1-Cre to CβA-FLEx plasmid. Consistent with the idea that labelling accurately reflects the promoter driving Cre expression, the proportion of GFP^{+} and TdTomato^{+} cells was stable across a range of plasmid ratios. A plasmid ratio of 1:1 (shaded blue) was used for the functional and morphological studies.). Error bars represent standard error of the mean. Source data are provided as a Source Data file.

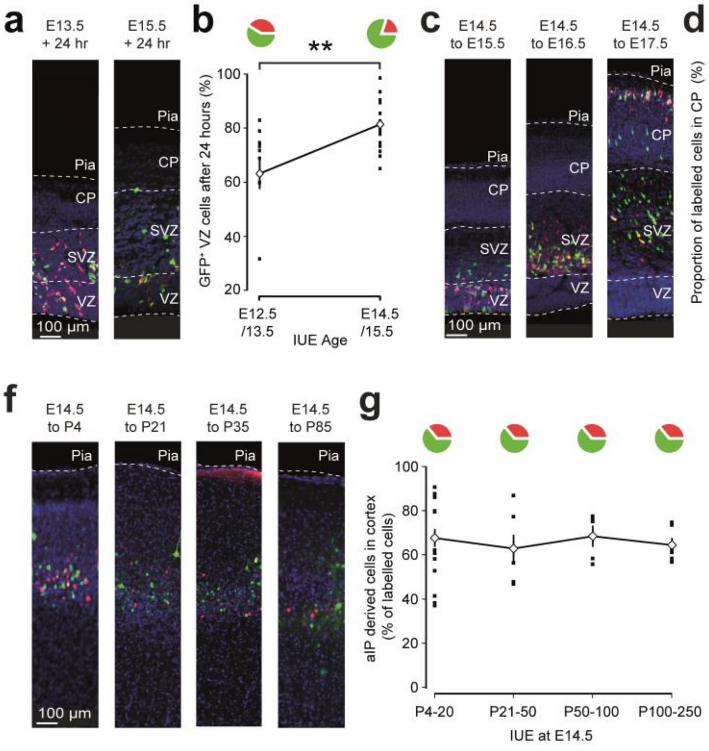

Supplementary Figure 3 aIPs represent a significant progenitor population in the embryonic VZ and the labeling of post-mitotic neurons remains stable.

(**a**) Tα1-Cre and CβA-FLEx plasmids were delivered by IUE at different embryonic ages (E12.5 to E15.5) and imaged 24h later. (**b**) Consistent with previous findings [1, 2], 24h after IUE revealed that GFP^+ aIPs represent a significant progenitor population in the embryonic mouse VZ. At this 24h time point, aIPs were defined as those cells within the VZ that expressed GFP (and could also still express some TdTomato). As has been reported [1], the aIP population was most prevalent during later stages of corticogenesis ($p = 0.002$, Mann-Whitney U test; n = 10 and 18 animals at E12.5 - E13.5 and E15.5 - E16.5, respectively). (**c**) GFP^+ and $TdTomato^+$ cells were imaged in embryonic cortex at 1d, 2d or 3d after IUE at E14.5. (**d**) Consistent with the migration of labeled post-mitotic neurons, the proportion of all fluorescently labeled cells that were located within the cortical plate (CP) showed a significant and progressive increase during embryonic development ($p = 0.0001$, Kruskal-Wallis test; n = 11, 7 and 9 animals at 1d, 2d and 3d after IUE at E14.5). (**e**) In support of the idea that the majority of Cre-mediated recombination of the reporter plasmid occurred within 24 h following IUE, the proportion of GFP^+ aIP-derived neurons in the CP remained stable during embryonic development ($p = 0.60$, Kruskal-Wallis test; n = 11, 7 and 9 animals at 1d, 2d and 3d after IUE at E14.5). (**f**) GFP^+ and $TdTomato^+$ cortical neurons were imaged across a range of postnatal ages after IUE at E14.5. (**g**) The proportion of GFP^+ aIP-derived cortical neurons did not change significantly across a wide range of postnatal ages ($p = 0.94$, Kruskal-Wallis test, n = 16, 6, 5 and 6 animals at P4-20, P21-50, P50-100 and P100-250 after IUE at E14.5), consistent with the idea that there was no further recombination of the reporter plasmid postnatally.). Error bars represent standard error of the mean. Source data are provided as a Source Data file.

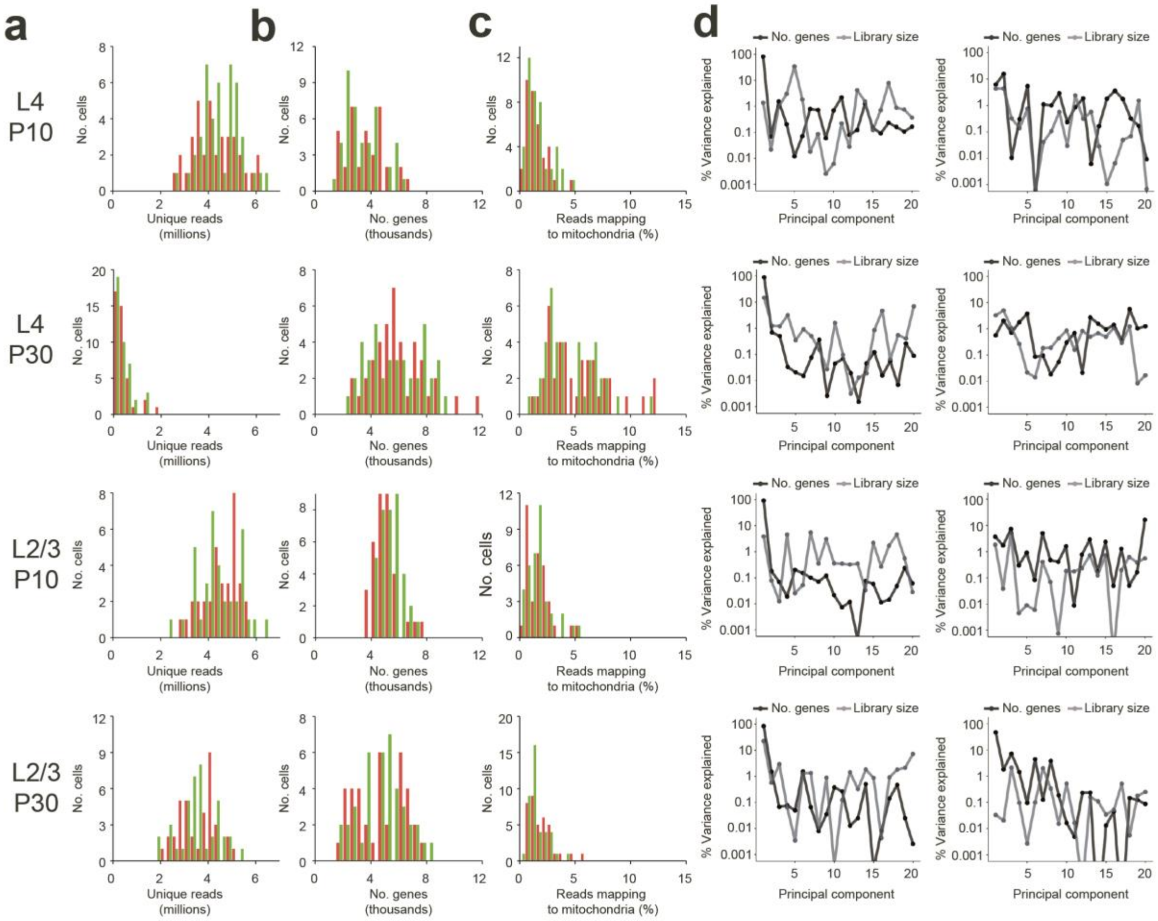

Supplementary Figure 4 Quality control data for single-cell RNA-seq analysis of excitatory cortical neurons derived from embryonic progenitor pools. For the different cortical layers and ages of cell collection (top to bottom), comparable data was obtained for the batches of aIP- (green) and OP-derived (red) cells in terms of (**a**) the number of unique reads per cell, (**b**) the number of genes detected per cell and (**c**) the proportion of mitochondrial reads per cell. (**d**) The results of data normalisation are shown for the same batches of cells as in 'A-C'. Plots show the correlation between the total number of reads (grey) and the number of genes detected (black), with each principal component after normalisation with *scran* (left) and following normalisation with *RUVSeq* (right).). Error bars represent standard error of the mean. Source data are provided as a Source Data file.

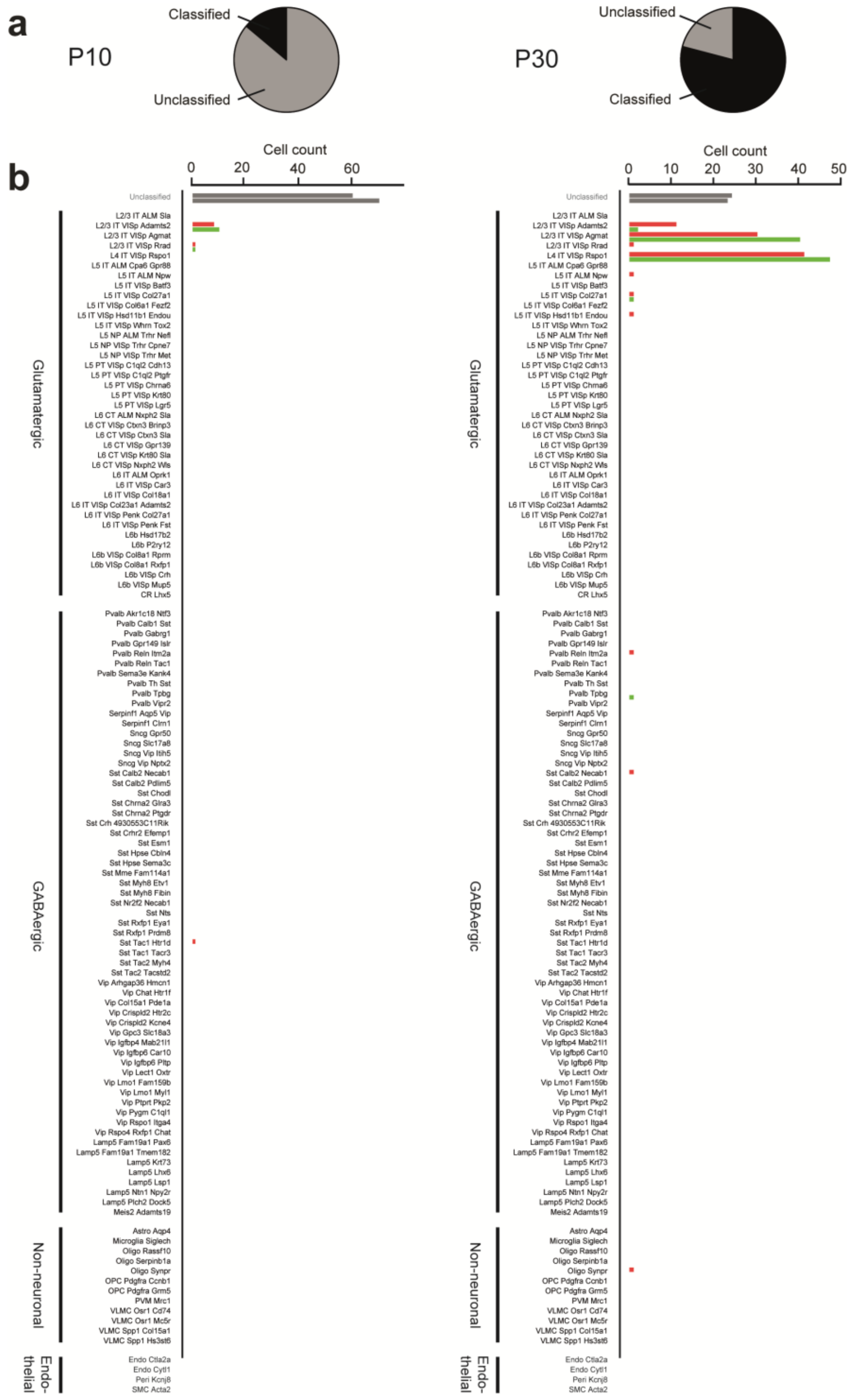

Supplementary Figure 5   Success rates for cell classification by single-cell RNA-seq. (**a**) Pie charts summarise the proportion of cells at P10 (left) and P30 (right) that were successfully classified against the dataset from [3]. **(b)** Histograms show aIP- (green) and OP-derived (red) cells classified at P10 (left) and P30 (right), against each of the cell classes investigated. Cells not assigned to one of the cell classes were labelled as ‘unclassified’ (grey).). Error bars represent standard error of the mean. Source data are provided as a Source Data file.

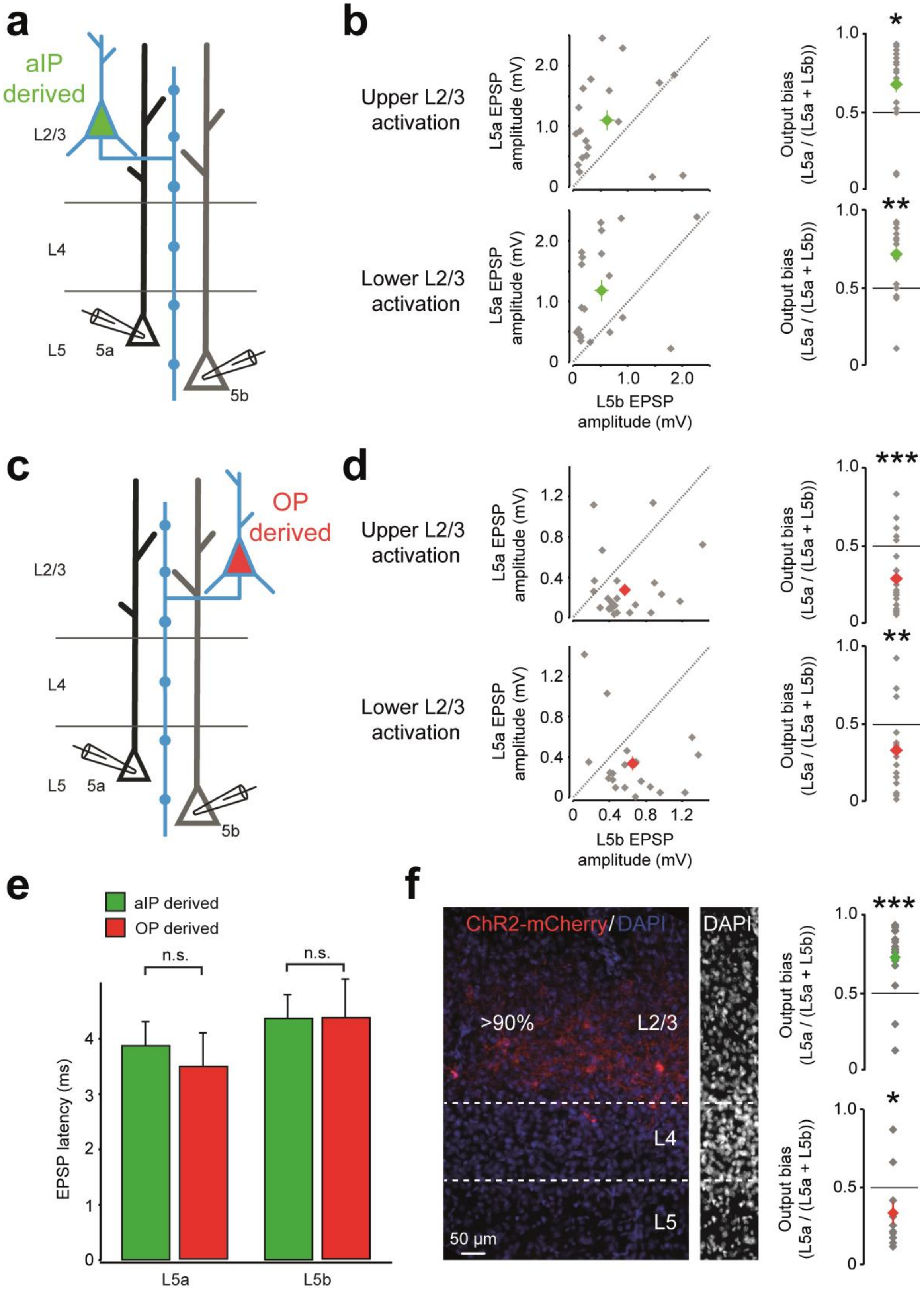

a
aIP derived
L2/3
L4
L5
5a
5b
b
Upper L2/3 activation
Lower L2/3 activation
L5a EPSP amplitude (mV)
L5b EPSP amplitude (mV)
Output bias (L5a / (L5a + L5b))
c
OP derived
d
e
aIP derived
OP derived
n.s.
EPSP latency (ms)
L5a
L5b
f
ChR2-mCherry/ DAPI
DAPI
>90%
50 μm

Supplementary Figure 6 Progenitor-associated differences in translaminar connectivity are evident across L2/3.

(**a**) L2/3 ChR2 fibers originating from aIPs were activated by delivering brief (1 ms), focal flashes of blue laser light over L2/3, whilst performing simultaneous whole-cell patch-clamp recordings from pairs of L5 pyramidal neurons, one of which was in L5a and the other in L5b. (**b**) Regardless of whether the light flash was delivered to the upper half of L2/3 (top), or the lower half of L2/3 (bottom), the aIP-derived pyramidal neurons showed a bias to drive L5a over L5b (upper output bias: 0.68 +/- 0.06, p = 0.013 and lower output bias: 0.71 +/- 0.05, p = 0.003, Wilcoxon signed rank test, n = 19 and 20 pairs, respectively). (**c**) In a separate set of experiments, L2/3 ChR2 fibers originating from OPs were activated whilst performing simultaneous whole-cell patch-clamp recordings from pairs of L5a and L5b pyramidal neurons. (**d**) The bias for OP-derived L2/3 pyramidal neurons to preferentially excite L5b pyramidal neurons was evident regardless of whether the light flash was delivered to the upper half of L2/3 (top) or the lower half of L2/3 (bottom) (upper output bias: 0.29 +/- 0.04, p = 0.0004 and lower output bias: 0.34 +/- 0.0064, p = 0.002, Wilcoxon signed rank test, n = 25 and 22 pairs, respectively). **(e)** EPSP latency recorded in L5a and L5b neurons was comparable when activating the ChR2-exressing inputs from either aIP-derived or OP-derived neurons (p > 0.05 in all cases, t-test). (**f**) To establish that the biases were associated with L2/3 output, the analysis was restricted to tissue in which the IUE had labelled almost exclusively L2/3 neurons (i.e. >90% of the ChR2-expressing neurons were located within L2/3). For these data, the aIP-derived population still showed a significant bias in their output to L5a over L5b (output bias of 0.72 ± 0.06; p=0.006, Wilcoxon, n=16) and the OP-derived population showed a significant bias in their output to L5b (output bias of 0.33 ± 0.07; p=0.041, Wilcoxon, n=12).). Error bars represent standard error of the mean. Source data are provided as a Source Data file.

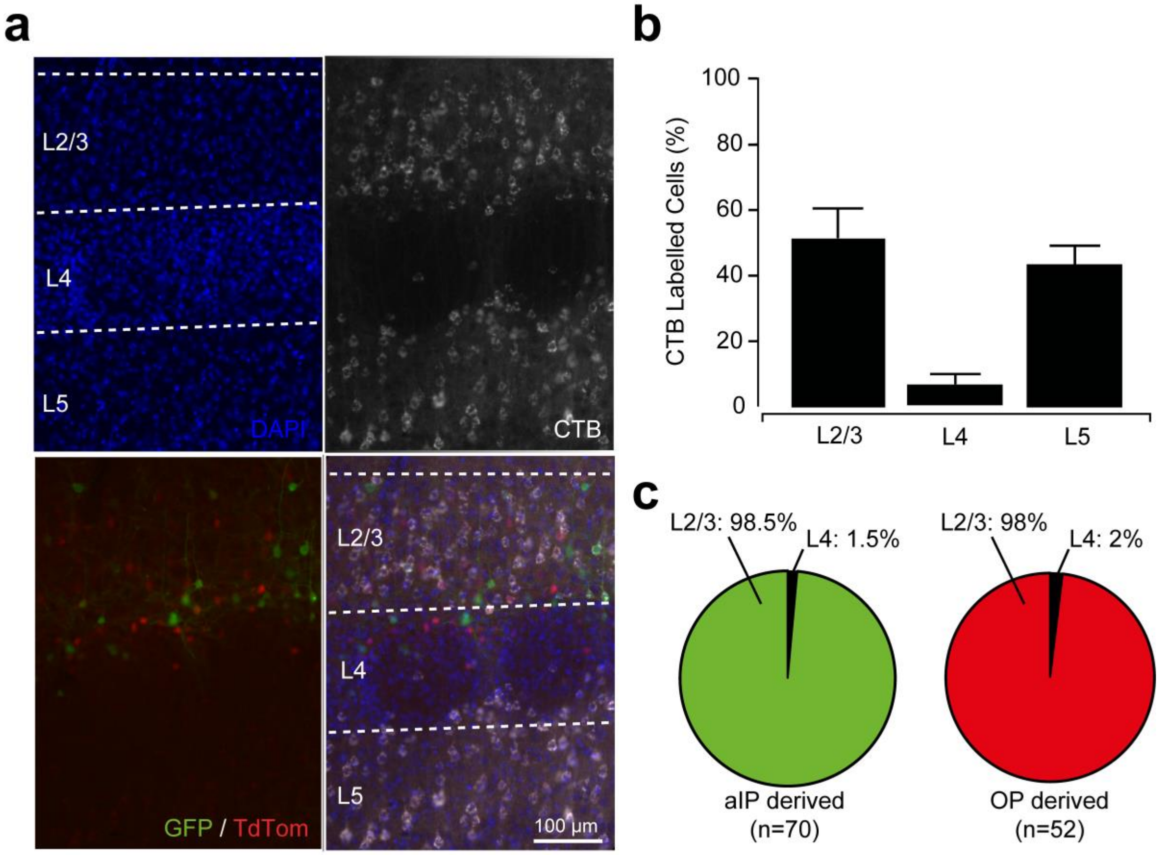

Supplementary Figure 7 L2/3 is the major source of callosal projections from neurons labelled by IUE.

(**a**) Adult mice that had undergone IUE received an injection of the retrograde tracer, Choleratoxin β-subunit (CTB), into the primary somatosensory cortex of the hemisphere contralateral to that which had been electroporated. DAPI staining was used to determine cortical layers and counts were made of all S1 callosally-projecting neurons labelled with CTB. Of these retrogradely-labelled neurons, the aIP- and OP-derived populations were determined by the expression of GFP or TdTomato, respectively. (**b**) Bar plot shows that the majority of all callosally-projecting neurons reside in L2/3 and L5 (n = 3 animals). (**c**) For the aIP- and OP-derived populations labelled by IUE, the callosally-projecting neurons were almost exclusively located in L2/3.). Error bars represent standard error of the mean. Source data are provided as a Source Data file.

Supplementary Table 1: Intrinsic membrane properties

Layer 4 spiny stellate neurons:

	aIP derived	OP derived	p-value	Unlabelled
Resting membrane potential	-65.3 ± 0.7	-66.1 ± 0.7	0.26	-66.3 ± 0.7
Input resistance (MΩ)	131.1 ± 7.0	133.0 ± 8.4	0.99	121.6 ± 7.3
Spike threshold (mV)	-41.0 ± 0.6	-41.0 ± 0.6	0.52	-40.3 ± 0.9
Spike rate (500pA) (Hz)	32.5 ± 2.0	35.2 ± 1.9	0.27	33.7 ± 2.2
Spike rate (400pA) (Hz)	27.9 ± 1.8	31.6 ± 1.9	0.16	29.6 ± 2.3
Spike rate (300pA) (Hz)	21.5 ± 1.7	24.4 ± 1.6	0.22	22.8 ± 2.3
Spike rate (200pA) (Hz)	15.3 ± 1.3	17.1 ± 1.4	0.36	17.0 ± 1.9
Spike rate (100pA) (Hz)	8.0 ± 0.9	7.9 ± 1.0	0.85	8.4 ± 1.4
First ISI (ms)	13.8 ± 2.1	12.3 ± 2.2	0.22	14.9 ± 2.5
Second ISI (ms)	26.2 ± 3.2	20.2 ± 2.7	0.11	25.4 ± 3.4
Third ISI (ms)	36.9 ± 4.8	25.0 ± 2.7	0.11	31.4 ± 3.9
Fourth ISI (ms)	38.7 ± 4.8	28.5 ± 2.6	0.21	32.3 ± 2.5
First spike amplitude (mV)	53.9 ± 2.2	51.2 ± 2.1	0.16	48.7 ± 2.8
Second spike amplitude (mV)	41.5 ± 1.9	39.9 ± 1.9	0.54	41.3 ± 2.4
First spike duration (ms)	1.8 ± 0.1	1.8 ± 0.0	0.71	1.7 ± 0.1
Second spike duration (ms)	2.7 ± 0.2	2.5 ± 0.1	0.54	2.4 ± 0.1

Layer 2/3 pyramidal neurons:

	aIP derived	OP derived	p-value	Unlabelled
Resting membrane potential	-68.3 ± 0.6	-68.3 ± 0.7	0.89	-68.4 ± 0.6
Input resistance (MΩ)	99.3 ± 5.9	96.7 ± 6.6	0.95	101.8 ± 5.8
Spike threshold (mV)	-39.6 ± 1.4	-39.5 ± 0.9	0.49	-39.5 ± 0.6
Spike rate (500pA) (Hz)	28.8 ± 1.6	31.2 ± 2.2	0.66	28.7 ± 1.5
Spike rate (400pA) (Hz)	26.9 ± 1.9	27.7 ± 1.7	0.56	24.2 ± 1.3
Spike rate (300pA) (Hz)	20.6 ± 1.6	20.7 ± 1.8	0.94	18.4 ± 1.3
Spike rate (200pA) (Hz)	15.5 ± 1.4	13.9 ± 1.6	0.43	12.4 ± 1.2
Spike rate (100pA) (Hz)	10.7 ± 1.3	8.2 ± 1.3	0.19	7.2 ± 1.5
First ISI (ms)	23.2 ± 3.5	22.1 ± 4.1	0.65	22.6 ± 2.1
Second ISI (ms)	36.5 ± 3.9	33.6 ± 4.2	0.62	39.0 ± 3.5
Third ISI (ms)	45.7 ± 4.9	38.3 ± 4.0	0.38	42.7 ± 3.5
Fourth ISI (ms)	44.3 ± 6.5	42.1 ± 4.2	0.79	39.7 ± 2.1
First spike amplitude (mV)	51.1 ± 1.9	49.8 ± 2.1	0.73	48.3 ± 1.9
Second spike amplitude (mV)	42.9 ± 1.9	44.5 ± 1.9	0.72	43.7 ± 1.5
First spike duration (ms)	1.9 ± 0.1	1.8 ± 0.1	0.38	2.1 ± 0.1
Second spike duration (ms)	2.9 ± 0.1	2.9 ± 0.2	0.65	3.1 ± 0.1

Data are given as mean ± SEM, statistical comparsions by Mann-Whitney U test

Supplementary Table 1 Summary of intrinsic membrane properties for L4 spiny stellate neurons (top) and L2/3 pyramidal neurons (bottom) derived from different progenitor pools. Source data are provided as a Source Data file.

Supplementary Table 2: Synaptic response properties

Layer 4 spiny stellate neurons:

	aIP derived to aIP derived	aIP derived to OP derived	p-value	OP derived to OP derived	OP derived to aIP derived	p-value
Amplitude (mV)	0.78 ± 0.15	0.66 ± 0.33	0.15	0.67 ± 0.31	0.93 ± 0.22	0.31
Duration (ms)	161.90 ± 5.83	172.22 ± 34.31	0.86	114.74 ± 14.20	158.04 ± 23.46	0.13
Rise time (ms)	5.75 ± 0.50	6.63 ± 1.16	0.73	7.93 ± 1.53	6.39 ± 0.46	0.66
Decay time (ms)	75.20 ± 2.42	79.48 ± 17.10	0.86	49.02 ± 6.30	72.63 ± 11.47	0.09
Short term plasticity (2 vs 1)	0.60 ± 0.23	0.73 ± 0.07	0.52	0.72 ± 0.12	0.64 ± 0.10	0.77
Short term plasticity (3 vs 1)	0.52 ± 0.25	0.68 ± 0.09	0.52	0.60 ± 0.11	0.54 ± 0.06	0.95
Short term plasticity (4 vs 1)	0.43 ± 0.13	0.62 ± 0.09	0.27	0.55 ± 0.10	0.47 ± 0.08	0.44
Short term plasticity (5 vs 1)	0.44 ± 0.17	0.71 ± 0.12	0.38	0.54 ± 0.11	0.45 ± 0.09	0.44
Short term plasticity (6 vs 1)	0.57 ± 0.22	0.65 ± 0.09	1.00	0.62 ± 0.08	0.43 ± 0.08	0.08

Layer 2/3 pyramidal neurons:

	aIP derived to aIP derived	aIP derived to OP derived	p-value	OP derived to OP derived	OP derived to aIP derived	p-value
Amplitude (mV)	0.57 ± 0.15	0.70 ± 0.18	0.66	0.90 ± 0.15	0.52 ± 0.21	0.07
Duration (ms)	131.04 ± 17.38	118.72 ± 9.55	0.61	110.21 ± 14.14	95.76 ± 12.06	0.26
Rise time (ms)	6.78 ± 0.80	5.98 ± 0.78	0.20	5.36 ± 0.67	6.55 ± 0.59	0.53
Decay time (ms)	58.74 ± 8.74	53.38 ± 4.54	0.47	49.74 ± 7.08	41.33 ± 6.08	0.26
Short term plasticity (2 vs 1)	0.80 ± 0.19	0.76 ± 0.09	0.97	0.76 ± 0.15	0.75 ± 0.15	0.93
Short term plasticity (3 vs 1)	0.74 ± 0.27	0.70 ± 0.09	0.79	0.59 ± 0.12	0.71 ± 0.17	0.93
Short term plasticity (4 vs 1)	0.37 ± 0.11	0.61 ± 0.08	0.21	0.54 ± 0.07	0.70 ± 0.13	0.43
Short term plasticity (5 vs 1)	0.50 ± 0.10	0.59 ± 0.08	0.52	0.48 ± 0.10	0.60 ± 0.11	0.93
Short term plasticity (6 vs 1)	0.46 ± 0.11	0.64 ± 0.09	0.34	0.40 ± 0.07	0.58 ± 0.12	0.54

Data are given as mean ± SEM, statistical comparsions by Mann-Whitney U test

Supplementary Table 2 Summary of synaptic response properties for different connection types between L4 spiny stellate neurons (top) and L2/3 pyramidal neurons (bottom). Source data are provided as a Source Data file.

References

1. Gal, J.S., *et al.* Molecular and morphological heterogeneity of neural precursors in the mouse neocortical proliferative zones. *J Neurosci* **26**, 1045-1056 (2006).
2. Stancik, E.K., Navarro-Quiroga, I., Sellke, R. & Haydar, T.F. Heterogeneity in ventricular zone neural precursors contributes to neuronal fate diversity in the postnatal neocortex. *J Neurosci* **30**, 7028-7036 (2010).
3. Tasic, B., *et al.* Shared and distinct transcriptomic cell types across neocortical areas. *Nature* **563**, 72-78 (2018).
