# Supplementary material for: Embryonic progenitor pools generate diversity in fine-scale excitatory cortical subnetworks
Source: Nat Commun. 2019 Nov 19;10:5224. doi: 10.1038/s41467-019-13206-1 (PMC6863870; doi:10.1038/s41467-019-13206-1)
Supplement: Supplementary file 3 — Reporting Summary [file 41467_2019_13206_MOESM3_ESM.pdf]

nature research

Corresponding author(s): Colin J. Akerman

Last updated by author(s): Aug 23, 2019

Reporting Summary

Nature Research wishes to improve the reproducibility of the work that we publish. This form provides structure for consistency and transparency in reporting. For further information on Nature Research policies, see Authors & Referees and the Editorial Policy Checklist .

Statistics

For all statistical analyses, confirm that the following items are present in the figure legend, table legend, main text, or Methods section.

n/a	Confirmed	
☐	☒	The exact sample size (n) for each experimental group/condition, given as a discrete number and unit of measurement
☐	☒	A statement on whether measurements were taken from distinct samples or whether the same sample was measured repeatedly
☐	☒	The statistical test(s) used AND whether they are one- or two-sided<br>*Only common tests should be described solely by name; describe more complex techniques in the Methods section.*
☒	☐	A description of all covariates tested
☐	☒	A description of any assumptions or corrections, such as tests of normality and adjustment for multiple comparisons
☐	☒	A full description of the statistical parameters including central tendency (e.g. means) or other basic estimates (e.g. regression coefficient) AND variation (e.g. standard deviation) or associated estimates of uncertainty (e.g. confidence intervals)
☐	☒	For null hypothesis testing, the test statistic (e.g. F, t, r) with confidence intervals, effect sizes, degrees of freedom and P value noted<br>*Give P values as exact values whenever suitable.*
☒	☐	For Bayesian analysis, information on the choice of priors and Markov chain Monte Carlo settings
☒	☐	For hierarchical and complex designs, identification of the appropriate level for tests and full reporting of outcomes
☒	☐	Estimates of effect sizes (e.g. Cohen's d, Pearson's r), indicating how they were calculated

Our web collection on statistics for biologists contains articles on many of the points above.

Software and code

Policy information about availability of computer code

Data collection	All commercial software for data collection is detailed in the manuscript. These included: pClamp9 (Molecular Devices, RRID:SCR_011323) and WinWCP software (University of Strathclyde, UK, RRID:SCR_014713).
Data analysis	All commercial software for data analysis is detailed in the manuscript. These included SPSS 17.0 (IBM SPSS statistics, RRID:SCR_002865), GraphPad Prism version 5.0 (GraphPad Software, RRID:SCR_002798), MATLAB (RRID:SCR_001622) and Igor Pro (Wavemetrics, RRID:SCR_000325). Custom analysis code is made available at https://github.com/jscaber/cgat-proj057.

For manuscripts utilizing custom algorithms or software that are central to the research but not yet described in published literature, software must be made available to editors/reviewers. We strongly encourage code deposition in a community repository (e.g. GitHub). See the Nature Research guidelines for submitting code & software for further information.

Data

Policy information about availability of data

All manuscripts must include a data availability statement. This statement should provide the following information, where applicable:

- Accession codes, unique identifiers, or web links for publicly available datasets
- A list of figures that have associated raw data
- A description of any restrictions on data availability

Provide your data availability statement here.

Field-specific reporting

Please select the one below that is the best fit for your research. If you are not sure, read the appropriate sections before making your selection.

[x] Life sciences [ ] Behavioural & social sciences [ ] Ecological, evolutionary & environmental sciences

For a reference copy of the document with all sections, see nature.com/documents/nr-reporting-summary-flat.pdf

Life sciences study design

All studies must disclose on these points even when the disclosure is negative.

Sample size	Sample sizes were chosen based on accepted standards in the field. These are sufficient to generate meaningful conclusions given biologically relevant effect sizes and typical data variance for the measures used.
Data exclusions	No data was excluded.
Replication	Three approaches were taken with regards to reproducibility. Firstly, intrinsic electrophysiological properties of both aIP- and OP-derived neurons were recorded separately by two of the authors (TE and SVA) and both data sets confirmed the observation that intrinsic electrophysiological properties are similar between SNP- and OP-derived neurons. Secondly, progenitor-based differences in intralaminar synaptic connectivity were detected in two separate cortical layers. A bias in intralaminar connectivity was initially observed within layer L2/3 of cortex and, at this point, intralaminar connectivity was then assessed within layer 4 of cortex and progenitor-based differences were replicated. Thirdly, progenitor-based differences in extralaminar synaptic connectivity were observed in two pathways: ipsilateral translaminar connections from layer 2/3 to layer 5 and contralateral translaminar connections from layer 2/3 to layer 5.
Randomization	All patch clamp recordings were performed randomly, in the sense that aIP- and OP-derived neurons were both assessed in individual recording sessions, in randomized configurations, and using different recording headstages. This approach avoided potential biases in cellular and circuit properties that could be associated with individual animals, the time of recording or equipment.
Blinding	Wherever possible, we used blinding during data analysis. Most experiments involved large data sets and analysis employed automated, or semi-automated computer scripts, in which the experimenter was blinded to cell type or brain region. For example, the analysis of all intracellular recording data (electrophysiological properties and synaptic connectivity) was performed blinded to a neuron's progenitor type of origin, and anatomical data was analyzed blinded to injection location and progenitor of origin.

Reporting for specific materials, systems and methods

We require information from authors about some types of materials, experimental systems and methods used in many studies. Here, indicate whether each material, system or method listed is relevant to your study. If you are not sure if a list item applies to your research, read the appropriate section before selecting a response.

Materials & experimental systems

n/a	Involved in the study	
[ ]	[x]	Antibodies
[x]	[ ]	Eukaryotic cell lines
[x]	[ ]	Palaeontology
[ ]	[x]	Animals and other organisms
[x]	[ ]	Human research participants
[x]	[ ]	Clinical data

Methods

n/a	Involved in the study	
[x]	[ ]	ChIP-seq
[x]	[ ]	Flow cytometry
[x]	[ ]	MRI-based neuroimaging

Antibodies

Antibodies used	All relevant details are in the Methods section of the manuscript.
Validation	Manufacturer details are in the Methods section of the manuscript.

Animals and other organisms

Policy information about studies involving animals; ARRIVE guidelines recommended for reporting animal research

Laboratory animals	All relevant details are in the Methods section of the manuscript.
Wild animals	*Provide details on animals observed in or captured in the field; report species, sex and age where possible. Describe how animals were caught and transported and what happened to captive animals after the study (if killed, explain why and describe method; if released, say where and when) OR state that the study did not involve wild animals.*

Field-collected samples	*For laboratory work with field-collected samples, describe all relevant parameters such as housing, maintenance, temperature, photoperiod and end-of-experiment protocol OR state that the study did not involve samples collected from the field.*
Ethics oversight	All experiments were carried out in accordance with the UK Animals (Scientific Procedures) Act (1986).

Note that full information on the approval of the study protocol must also be provided in the manuscript.
