# Supplementary material for: Embryonic progenitor pools generate diversity in fine-scale excitatory cortical subnetworks
Source: Nat Commun. 2019 Nov 19;10:5224. doi: 10.1038/s41467-019-13206-1 (PMC6863870; doi:10.1038/s41467-019-13206-1)
Supplement: Supplementary file 2 — Peer Review [file 41467_2019_13206_MOESM2_ESM.pdf]

Reviewers' comments:

Reviewer #1 (Remarks to the Author):

This is an excellent manuscript systematically comparing the properties of aIP-derived versus OP-derived excitatory neurons in superficial layers of mouse S1 cortex. Experiments are all expertly executed. Properties assayed include single cell molecular profiles, morphology, intrinsic firing, local connectivity, long distance projections and long-distance cortico-cortical connectivity. While many properties were not distinguishable, important differences were observed in the molecular clusters occupied, in intralaminar connectivity, and in layer 5 cell types targeted by callosal connections to the contralateral S1. The manuscript is generally well written and the results are very interesting. Nearly all results are solid and have appropriate controls where needed. As noted below, however, there are potentially problems with the analysis of local connections from aIP or OP cells onto layer 5 neurons. The caveats mentioned in detail below should be highlighted in the revised manuscript and discussed.

This manuscript describes studies of the properties of layer 2/3 and layer 4 excitatory neurons of mouse somatosensory cortex and comparing neurons derived from apical intermediate progenitors (aIP-derived) versus from other progenitors (OP-derived). Comparisons are made between populations simultaneously labeled in the same animals by in utero electroporation at E14.5, resulting in labeling of neurons in upper layer 4 and lower layer 2/3. Use of a Tα1-Cre construct and a CβA-FLEx reporter construct results in GFP expression in aIPs and RFP in OPs.

These populations have largely overlapping laminar distributions but with some laminar differences. Single cell transcriptomics of P30 cells reveals that layer 4 cells fall into the previously described class of layer 4 excitatory neurons. Layer 2/3 cells differ significantly between the populations with aIPs being more uniform and 94% classified into a single cluster called Agmat (of 3 previously identified) and the remainder in one other cluster (Adamts). The OPs also fall primarily into the Agmat cluster (64%) with 24% Adamts and 11% Rrad. Intracellular recording and dye fills in brain slices revealed the expected spiny stellate morphologies in layer 4 and pyramidal morphologies in layer 2/3 as well as firing properties that were indistinguishable between aIPs and OPs.

Studies of the intralaminar connectivity between cell types yielded the most interesting results. Within layer 4, connections from aIP to other aIP are under-represented (only 3.1%) compared to all other combinations that have >10% rate of connectivity. An even more striking bias was observed for local connectivity within layer 2/3, with aIP to OP connections being over represented relative to all other combinations. Good controls rule out factors other than cell type that might have contributed to these results.

Studies of local connections from aIP versus OP neurons onto layer 5 neurons were not as cleanly interpretable. In utero electroporation was again used along with the Tα1-Cre construct, but this time combined with a Cre-ON or Cre-OFF ChR2-mCherry construct to express in aIPs or OPs. As noted above and clear from figure 6, this results in expression in both layer 4 and layer 2/3 cells. But there is presumably a higher proportion of layer 2/3 cells that are OP-derived and a higher proportion of layer 4 cells that are aIP derived (see also Figure 1). Thus, differences in responses measured in other neurons following photoactivation might be due to either the laminar differences or to the cell type differences, confounding clear interpretation. The results are presented as if there is no expression in layer 4 and simply describe photoactivation of “axons from layer 2/3 neurons”. Cell pairs in layer 5A and 5B were recorded and it was found that activation of aIP neurons generated somewhat stronger responses in the layer 5A than the layer 5B neuron (mean ratio of 0.67), but following activation of OPs the bias was reversed. The author’s interpretation of these results is likely flawed. Because it is highly likely that layer 4 neurons were activated and there is a higher proportion of aIP-derived layer 4 neurons, the different ratios observed could reflect stronger connections from layer 4 onto layer 5A versus 5B cells and/or stronger connections from layer 2/3 onto layer 5B cells. Note that even if light is restricted to layer 2/3, layer 4 cells send axons into layer 2/3 that can be activated. There is also

concern that responses measured might reflect polysynaptic activation rather than direct connectivity.

Similar methods were used to photoactivate axons from aIP or OP-derived neurons in the contralateral S1 cortex while recording from layer 5A/5B pyramid pairs. Here the results were similar to those for the local connectivity with stronger responses in the 5A cell following aIP axon activation (ratio of 0.68) and stronger responses in the layer 5B cell following OP axon activation (ratio of 0.28). These results are stronger than those for the intralaminar local connectivity insofar as layer 4 neurons probably do not make projections to the contralateral hemisphere and could not have contributed to the measured responses. Reporting the proportion of retrograde cTB labeled cells in layer 4 following injections in the contralateral S1 would reinforce this.

Finally , retrograde labeling studies show no differences in the proportions of aIP versus OP cells that project from S1 to S2, M1 or contralateral S1.

Minor points/clarification suggestions:

"We labeled the progenitor populations using in utero electroporation (IUE) of a Tα1-Cre construct and a CβA-FLEx reporter construct…" It would be helpful if the text here and/or in the figure legend described this construct in more detail. What is expressed from this construct and under what conditions? Then what color will be expressed in the aIP versus the OP neurons? There is not even any mention here of the fact that the construct drives expression of fluorescent proteins.

"… both populations were distributed vertically and horizontally throughout L4 and L2/3 (L4: 72% aIP-derived, 28% OP-derived; L2/3: 57% aIP-derived, 43% OPderived; Fig. 1e, f)." This is not an accurate description. Figure 1 shows expression that is restricted to a narrow laminar zone including the top of layer 4 and the bottom of layer 2/3. In addition to the quantification provided, it would be helpful to report what proportion of the OP-derived neurons are in layer 4 vs. layer 2/3 and also what proportion of aIP derived neurons are in each layer.

It is important that the layer 2/3, layer 4 border be accurately delineated. How was this done? The blue DAPI stain that should distinguish layer 4 is not visible in Figure 1d. A photograph that clearly shows the change in cell density that should be apparent for layer 4 of S1 cortex should be included in figure 1.

Reviewer #2 (Remarks to the Author):

In this manuscript, Ellender and colleagues use in utero electroporation to label different types of progenitor cells ("apical intermediate progenitors –aIPs"; and "other progenitors – OPs") in the developing dorsal telencephalon of mice. Next, the authors analyze the contribution of these different progenitor types for the generation of layer 4 and layer 2/3 neurons. Using targeted single-cell RNA sequencing, the authors show that aIPs and OPs give rise to different classes of transcriptionally-defined glutamatergic neurons. Next, using simultaneous patch clamp recordings and optogenetics, the authors show that aIP- and OP-derived neurons exhibit systematic biases in both intra-laminar and extra-laminar monosynaptic connectivity. These findings are extremely interesting and shed light on our understanding of how progenitor cell heterogeneity during development may contribute to the variety of neuronal cell types in the mammalian neocortex. However, I have some major concerns about the classification of progenitor types and the results involving single-cell RNAseq, which are detailed below:

1) The authors use Ta1 promoter to label progenitor cells that are classified as aIPs. However, Ta1 is likely active in every neurogenic IPs, including basal progenitors lacking both apical and basal

processes. In fact, it is possible to observe these cells in the FigS1. Moreover, there is a significant fraction (25%) of "aIPs" with basal process (FigS1). Thus, I feel that the authors must discuss their data in a less dogmatic way, avoiding the use of the term aIPs. They could, for example, use Ta1-expressing progenitors. Alternatively, the authors must use unambiguous strategies to exclusively fate-map aIPs-derived neurons.

2) The single-cell RNAseq data show that the Agmat+ population is larger than the Adamts2+ one, which in turn is larger than Rrad2+ population (Tasic et al., 2018). The author compare the proportion of these neuronal subtypes amongst "aIP"- and "OP"-derived neurons. I suggest that the authors calculate the probability of a neuron to become Agmat+, Adamts2+ or Rrad2+ in the general population (based on Tasic et al., 2018) and compare that to the the probability of a neuron derived from Ta1- or other-progenitors to adopt the same fate, as this may better indicate a fate bias.

3) The authors must consider the possibility that all biases suggested in their experiments (Figs 2, 4-7) are age- and not progenitor type-dependent. In fact, one could expect that aIP-derived cells will become post-mitotic neurons before OP-derived ones and this could explain the different neuronal fates. Performing some experiments at different time-points could easily test this.

Minor points:

1) The proportion of GFP+ and TdTom+ neurons in L4 and L2/3 must be described as mean ± s.e.m

2) For all experiments, the number of experimental units (animals) and independent experiments (pregnant females) must be provided.

3) In the discussion, the authors could speculate about the possible explanations for the different potentials of Ta1-expressing and other progenitors.

Reviewer #3 (Remarks to the Author):

Different progenitor subtypes contribute neurons during mammalian brain development. Ellender et al investigate the identity of neurons generated from the specific class of short/intermediate apical progenitors (aIP). Using a previously described approach to acutely label aIP and their progeny by in utero electroporation, the authors describe the neurons resulting from them while comparing this population to those neurons generated from other types of progenitors (OP). Transcriptomic, electrophysiology and connectomic is used to assess neurons in different cortical layers and brain regions showing a connectivity bias characterizing aIP.

The study is novel and interesting. Notably, an elegant combination of highly sophisticated techniques is used to address this question. I found this truly impressive. The manuscript is remarkably clear and the data of high quality.

I only have one criticism that I expect the authors to discuss. This is that the population of "other progenitors" OP is, per se, also heterogenous and primarily made by apical radial glia (RG) and SVZ, basal progenitors (BP). By labeling both together, the authors could not assess whethe aIP-derived neurons are similar (or different) to the former RG, the latter BP, or neither of the two making aIP unique.

I fully appreciate that addressing this point experimentally will go well beyond the purpose of the current study and better left for future work. Yet, the authors should dedicate part of the discussion to objectively and fairly address this main limitation of their work.

Response to Reviewers' comments
"Embryonic progenitor pools generate diversity in fine-scale excitatory cortical subnetworks", by Ellender et al.
(manuscript number: NCOMMS-19-13865)

Reviewer #1:

This is an excellent manuscript systematically comparing the properties of aIP-derived versus OP-derived excitatory neurons in superficial layers of mouse S1 cortex. Experiments are all expertly executed. Properties assayed include single cell molecular profiles, morphology, intrinsic firing, local connectivity, long distance projections and long-distance cortico-cortical connectivity. While many properties were not distinguishable, important differences were observed in the molecular clusters occupied, in intralaminar connectivity, and in layer 5 cell types targeted by callosal connections to the contralateral S1. The manuscript is generally well written and the results are very interesting. Nearly all results are solid and have appropriate controls where needed. As noted below, however, there are potentially problems with the analysis of local connections from aIP or OP cells onto layer 5 neurons. The caveats mentioned in detail below should be highlighted in the revised manuscript and discussed.

We were very pleased to read that the Reviewer describes our manuscript as "excellent" and that the experiments are "expertly executed".

This manuscript describes studies of the properties of layer 2/3 and layer 4 excitatory neurons of mouse somatosensory cortex and comparing neurons derived from apical intermediate progenitors (aIP-derived) versus from other progenitors (OP-derived). Comparisons are made between populations simultaneously labeled in the same animals by in utero electroporation at E14.5, resulting in labeling of neurons in upper layer 4 and lower layer 2/3. Use of a Tα1-Cre construct and a CβA-FLEx reporter construct results in GFP expression in aIPs and RFP in OPs. These populations have largely overlapping laminar distributions but with some laminar differences. Single cell transcriptomics of P30 cells reveals that layer 4 cells fall into the previously described class of layer 4 excitatory neurons. Layer 2/3 cells differ significantly between the populations with aIPs being more uniform and 94% classified into a single cluster called Agmat (of 3 previously identified) and the remainder in one other cluster (Adamts). The OPs also fall primarily into the Agmat cluster (64%) with 24% Adamts and 11% Rrad. Intracellular recording and dye fills in brain slices revealed the expected spiny stellate morphologies in layer 4 and pyramidal morphologies in layer 2/3 as well as firing properties that were indistinguishable between aIPs and OPs. Studies of the intralaminar connectivity between cell types yielded the most interesting results. Within layer 4, connections from aIP to other aIP are under-represented (only 3.1%) compared to all other combinations that have >10% rate of connectivity. An even more striking bias was observed for local connectivity within layer 2/3, with aIP to OP connections being over represented relative to all other combinations. Good controls rule out factors other than cell type that might have contributed to these results.

Studies of local connections from aIP versus OP neurons onto layer 5 neurons were not as cleanly interpretable. In utero electroporation was again used along with the Tα1-Cre construct, but this time combined with a Cre-ON or Cre-OFF ChR2-mCherry construct to express in aIPs or OPs. As noted above and clear from figure 6, this results in expression in

both layer 4 and layer 2/3 cells. But there is presumably a higher proportion of layer 2/3 cells that are OP-derived and a higher proportion of layer 4 cells that are aIP derived (see also Figure 1). Thus, differences in responses measured in other neurons following photoactivation might be due to either the laminar differences or to the cell type differences, confounding clear interpretation. The results are presented as if there is no expression in layer 4 and simply describe photoactivation of "axons from layer 2/3 neurons". Cell pairs in layer 5A and 5B were recorded and it was found that activation of aIP neurons generated somewhat stronger responses in the layer 5A than the layer 5B neuron (mean ratio of 0.67), but following activation of OPs the bias was reversed. The author's interpretation of these results is likely flawed. Because it is highly likely that layer 4 neurons were activated and there is a higher proportion of aIP-derived layer 4 neurons, the different ratios observed could reflect stronger connections from layer 4 onto layer 5A versus 5B cells and/or stronger connections from layer 2/3 onto layer 5B cells. Note that even if light is restricted to layer 2/3, layer 4 cells send axons into layer 2/3 that can be activated. There is also concern that responses measured might reflect polysynaptic activation rather than direct connectivity.

The Reviewer raises the concern that the effects we observed for translaminar output to L5a and L5b might be due to differences in the relative laminar distribution of aIP-derived and OP-derived neurons. The Reviewer also raises the concern that there might be a differential contribution of polysynaptic activity. To address these concerns, we have conducted new experiments and performed further analyses on existing data.

Regarding the question of laminar distribution, we have addressed this by investigating translaminar connectivity in tissue where the *in utero* electroporation technique resulted in predominantly L2/3 labelled neurons (>90% of the labelled neurons residing within L2/3). Electroporation for these experiments was performed mostly at E14.5 (and occasionally at E15.5), but the assignment of plug time in the pregnant dams and the relative time of surgery would differ from animal to animal, which contributes to variance from litter to litter in terms of the pattern of expression across layer 2/3 and layer 4 of somatosensory cortex. Importantly, we find that when we limit our analysis to those sections in which we confirmed that more than 90% of labelled neurons were within L2/3 (as defined using DAPI labelling), we find that the key findings still hold. The aIP-derived population show a statistically significant bias in their output to L5a over L5b, while the OP-derived population show a statistically significant bias in their output to L5b over L5a. These data are now reported in new Supplemental Figure 6f, which is also described in the main results.

Regarding the question of polysynaptic activity, we deliberately used low levels of laser activation to minimise the possibility of polysynaptic activity, which is reflected in the range of EPSP amplitudes. We have now added a statement in the methods section to highlight this. Furthermore, the original version of the manuscript reported the synaptic delays for the L5a and L5b neurons for both the aIP-derived population and the OP-derived population. We have retained this text in the methods section, but now added a new Supplemental Figure 6e (and corresponding source datafile) showing that synaptic delays are comparable between the aIP-derived and OP-derived inputs to L5a and L5b. These additional data strengthen the conclusion that differences in the L5a and L5b responses reflect differential targeting from L2/3, and we thank the Reviewer for their suggestions.

Similar methods were used to photoactivate axons from aIP or OP-derived neurons in the contralateral S1 cortex while recording from layer 5A/5B pyramid pairs. Here the results were similar to those for the local connectivity with stronger responses in the 5A cell following aIP axon activation (ratio of 0.68) and stronger responses in the layer 5B cell following OP axon activation (ratio of 0.28). These results are stronger than those for the intralaminar local connectivity insofar as layer 4 neurons probably do not make projections

to the contralateral hemisphere and could not have contributed to the measured responses. Reporting the proportion of retrograde cTB labeled cells in layer 4 following injections in the contralateral S1 would reinforce this.

The Reviewer raises a related concern for the interpretation of the data for the contralateral projections from L2/3 to contralateral L5. We agree with the Reviewer's prediction that, based on previous literature, L4 neurons would not be expected to contribute significantly to the projection to S1 in the contralateral hemisphere. To investigate this ourselves, we have conducted new experiments to measure the proportion of L4 cells that are retrogradely labelled following a choleratoxin injection into contralateral S1. These new data are presented in new Supplementary Figure 7. In line with the Reviewer's prediction, we observe a very small proportion of L4 neurons that are retrogradely labelled. Furthermore, when we examined the proportion of aIP- and OP-derived neurons that are labelled following a choleratoxin injection into contralateral S1, we find that over 98% of these are L2/3 neurons for both aIP- and OP-derived cells. We thank the Reviewer for this suggestion and we are now able to make the stronger claim that our observed difference in contralateral L5a/L5b targeting is a feature of aIP and OP-derived L2/3 neurons.

Finally, retrograde labeling studies show no differences in the proportions of aIP versus OP cells that project from S1 to S2, M1 or contralateral S1.

Minor points/clarification suggestions:

"We labeled the progenitor populations using in utero electroporation (IUE) of a Tα1-Cre construct and a CβA-FLEx reporter construct…" It would be helpful if the text here and/or in the figure legend described this construct in more detail. What is expressed from this construct and under what conditions? Then what color will be expressed in the aIP versus the OP neurons? There is not even any mention here of the fact that the construct drives expression of fluorescent proteins.

We thank the Reviewer for encouraging us to provide more information about the constructs used to label progenitor-derived neurons. In the revised version of the manuscript, we now include information at the relevant point in the Results, which is partly repeated from the original Methods, but provides the details needed to fully understand the labelling strategy.

"... both populations were distributed vertically and horizontally throughout L4 and L2/3 (L4: 72% aIP-derived, 28% OP-derived; L2/3: 57% aIP-derived, 43% OPderived; Fig. 1e, f)." This is not an accurate description. Figure 1 shows expression that is restricted to a narrow laminar zone including the top of layer 4 and the bottom of layer 2/3. In addition to the quantification provided, it would be helpful to report what proportion of the OP-derived neurons are in layer 4 vs. layer 2/3 and also what proportion of aIP derived neurons are in each layer.

The Reviewer is correct that we could describe the Figure more accurately and so we have edited the text accordingly. In addition, we have performed the analysis suggested by the Reviewer and we now report that the proportion of OP-derived neurons in layer 4 is 36.7 ± 5.2% and 63.3 ± 5.2% in layer 2/3 and for aIP-derived neurons this in 37.5 ± 3.8% in layer 4 and 62.5 ± 3.8% in layer 2/3. These data are now reported in the results section of the main manuscript.

It is important that the layer 2/3, layer 4 border be accurately delineated. How was this done? The blue DAPI stain that should distinguish layer 4 is not visible in Figure 1d. A

photograph that clearly shows the change in cell density that should be apparent for layer 4 of S1 cortex should be included in figure 1.

Laminar borders were delineated from DAPI staining. We now add this important detail to the Methods section and include an illustrative example in revised Figure 1.

Reviewer #2:

In this manuscript, Ellender and colleagues use in utero electroporation to label different types of progenitor cells ("apical intermediate progenitors –aIPs"; and "other progenitors – OPs") in the developing dorsal telencephalon of mice. Next, the authors analyze the contribution of these different progenitor types for the generation of layer 4 and layer 2/3 neurons. Using targeted single-cell RNA sequencing, the authors show that aIPs and OPs give rise to different classes of transcriptionally-defined glutamatergic neurons. Next, using simultaneous patch clamp recordings and optogenetics, the authors show that aIP- and OP-derived neurons exhibit systematic biases in both intra-laminar and extra-laminar monosynaptic connectivity. These findings are extremely interesting and shed light on our understanding of how progenitor cell heterogeneity during development may contribute to the variety of neuronal cell types in the mammalian neocortex. However, I have some major concerns about the classification of progenitor types and the results involving single-cell RNAseq, which are detailed below:

1) The authors use Ta1 promoter to label progenitor cells that are classified as aIPs. However, Ta1 is likely active in every neurogenic IPs, including basal progenitors lacking both apical and basal processes. In fact, it is possible to observe these cells in the FigS1. Moreover, there is a significant fraction (25%) of "aIPs" with basal process (FigS1). Thus, I feel that the authors must discuss their data in a less dogmatic way, avoiding the use of the term aIPs. They could, for example, use Ta1-expressing progenitors. Alternatively, the authors must use unambiguous strategies to exclusively fate-map aIPs-derived neurons.

We agree with the Reviewer that the manuscript could be improved in how it describes the labelling strategy. We have therefore increased our description of IUE, expanded on how our data replicates published findings and moderated our wording. We cite evidence that the method targets actively dividing VZ progenitors and explain that, when we examine the earliest time point after DNA expression permits, our data replicate previous observations that GFP preferentially identifies aIPs that lack a basal process during metaphase, exhibit short ascending processes during their cell cycle, and represent a larger proportion of the VZ progenitor pool at E14.5 (Supplementary Figs 1 and 3). This is 24 hours after IUE and so may not precisely reflect the cells targeted at 0 hours. The Reviewer points out that whilst the majority of GFP^+ progenitors at this time classify morphologically as aIPs, the population includes a subset whose morphologies do not classify as aIPs. This is consistent with published work and we have revised the wording to explain that the labelling distinguishes a population of VZ progenitors that is enriched for aIPs, and which can be compared to OPs labelled at the same developmental stage in the same animal. Finally, we have added a discussion section on the strengths and limitations of the *in utero* labelling strategy. We point out that whilst the labelling allows us to ultimately identify mature neurons that were derived from progenitor pools, the method represents a developmental snapshot and provides little information on the subsequent lineage pathways taken by the progenitors or neurons.

2) The single-cell RNAseq data show that the Agmat+ population is larger than the Adamts2+ one, which in turn is larger than Rrad2+ population (Tasic et al., 2018). The

author compare the proportion of these neuronal subtypes amongst "aIP"- and "OP"-derived neurons. I suggest that the authors calculate the probability of a neuron to become Agmat+, Adamts2+ or Rrad2+ in the general population (based on Tasic et al., 2018) and compare that to the the probability of a neuron derived from Ta1- or other-progenitors to adopt the same fate, as this may better indicate a fate bias.

The Reviewer makes the interesting suggestion that, in addition to our existing statistical comparisons between neighbouring aIP-derived and OP-derived L2/3 neurons, it would also be informative to compare our data to the relative proportions of Agmat+, Adamts2+ and Rrad2+ L2/3 neurons derived from the larger dataset of Tasic et al., 2018. We have now conducted this new statistical analysis and find that aIP-derived neurons also represent a restricted output when compared to the general population ($p = 0.009$, Chi Square test). These new data are included in the revised Results section and we thank the Reviewer for this suggestion.

3) The authors must consider the possibility that all biases suggested in their experiments (Figs 2, 4-7) are age- and not progenitor type-dependent. In fact, one could expect that aIP-derived cells will become post-mitotic neurons before OP-derived ones and this could explain the different neuronal fates. Performing some experiments at different time-points could easily test this.

The Reviewer points out that the biases observed between aIP-derived and OP-derived neurons could result from age effects (i.e. time since becoming post-mitotic). This is an interesting point, to which we have now devoted attention in the discussion of the revised manuscript. As we write, differences in the exact birth-time of the neurons could play a role in determining the identity of the neurons and different connectivity patterns that we observe. Indeed, our data replicates previous evidence that within L2/3, the soma of aIP-derived neurons tend to reside somewhat further from the pial surface, which suggests a difference in the distribution of birth dates between the neurons ultimately derived from the two progenitor pools (Figure 1e). Age effects may therefore be interwoven with progenitor type and it would be challenging to unpick how these processes contribute. However, we also point out in our discussion that any effect of cell age would have to be reconciled with the fact that when we sampled neurons for transcriptomic analysis, or synaptic interconnectivity, we sampled neighbouring neurons that were matched in terms of their distance to the pial surface. As in the original version of the manuscript, data on this is provided in Figure 4d and Figure 5d. Thus, any age-effects would have to operate beyond the relative somatic position within cortex, perhaps as a result of different cohorts of cells also showing different migration patterns. We thank the Reviewer for encouraging us to discuss this idea within word limits.

Minor points:

1) The proportion of GFP+ and TdTom+ neurons in L4 and L2/3 must be described as mean ± s.e.m

This data is now reported in the text.

2) For all experiments, the number of experimental units (animals) and independent experiments (pregnant females) must be provided.

This information has now been added to the source data file.

3) In the discussion, the authors could speculate about the possible explanations for the different potentials of Ta1-expressing and other progenitors.

We have interpreted the Reviewer's phrase "different potentials" to mean that neurons derived from populations of progenitors can exhibit different properties (i.e. transcriptomic identities or synaptic connectivity patterns). Following the Reviewer's suggestion, we have extended our existing speculation in the discussion in two places: first, where we discuss why progenitors may be producing different transcriptomically-defined neuron types and second, the broader question of why progenitors may be producing different excitatory circuits in cortex.

Reviewer #3

Different progenitor subtypes contribute neurons during mammalian brain development. Ellender et al investigate the identity of neurons generated from the specific class of short/intermediate apical progenitors (aIP). Using a previously described approach to acutely label aIP and their progeny by in utero electroporation, the authors describe the neurons resulting from them while comparing this population to those neurons generated from other types of progenitors (OP). Transcriptomic, electrophysiology and connectomic is used to assess neurons in different cortical layers and brain regions showing a connectivity bias characterizing aIP.

The study is novel and interesting. Notably, an elegant combination of highly sophisticated techniques is used to address this question. I found this truly impressive. The manuscript is remarkably clear and the data of high quality.

We are delighted that the Reviewer agrees that our study is "novel", "interesting" and "truly impressive".

I only have one criticism that I expect the authors to discuss. This is that the population of "other progenitors" OP is, per se, also heterogenous and primarily made by apical radial glia (RG) and SVZ, basal progenitors (BP). By labeling both together, the authors could not assess whethe aIP-derived neurons are similar (or different) to the former RG, the latter BP, or neither of the two making aIP unique. I fully appreciate that addressing this point experimentally will go well beyond the purpose of the current study and better left for future work. Yet, the authors should dedicate part of the discussion to objectively and fairly address this main limitation of their work.

The Reviewer encourages us to reflect on the cell population that we define as "other progenitors". As the Reviewer correctly identifies, this is likely to comprise a heterogenous population of progenitors, particularly as development proceeds. We now address this important point in the revised discussion where we acknowledge that heterogeneity within OPs means that it is unknown which of these progenitor types contribute to the neuronal differences observed here. Equally, we cannot exclude that there are subtypes of aIPs, which future work may reveal can be distinguished with increasing resolution. We therefore point out the potential for future efforts in isolating defined progenitor subpopulations and characterising the connectivity of their neuronal progeny. We thank the Reviewer for encouraging us to highlight this exciting area.

REVIEWERS' COMMENTS:

Reviewer #1 (Remarks to the Author):

The authors have done an excellent job of addressing all of the concerns that I raised in the review of the original submission. The new analyses and descriptions are strong and convincing.

Reviewer #2 (Remarks to the Author):

All my concerns were satisfactorily addressed. I congratulate the authors for this elegant work supporting the notion that different progenitor types generate distinct neuronal classes, contributing to specific cortical subnetworks. It is a work of undeniable importance to our understanding of neocortical development.

Reviewer #3 (Remarks to the Author):

the authors have additionally revised my remaining comments satisfactorily hence i have no remaining concern about this nice work

Response to Reviewers' comments
"Embryonic progenitor pools generate diversity in fine-scale excitatory cortical subnetworks", by Ellender et al.
(manuscript number: NCOMMS-19-13865B)

Reviewer #1 (Remarks to the Author):
The authors have done an excellent job of addressing all of the concerns that I raised in the review of the original submission. The new analyses and descriptions are strong and convincing.
We thank the Reviewer for their positive feedback. There is nothing to address.

Reviewer #2 (Remarks to the Author):
All my concerns were satisfactorily addressed. I congratulate the authors for this elegant work supporting the notion that different progenitor types generate distinct neuronal classes, contributing to specific cortical subnetworks. It is a work of undeniable importance to our understanding of neocortical development.
We thank the Reviewer for their positive feedback. There is nothing to address.

Reviewer #3 (Remarks to the Author):
the authors have additionally revised my remaining comments satisfactorily hence i have no remaining concern about this nice work
We thank the Reviewer for their positive feedback. There is nothing to address.
